# Blocking Lysine Crotonylation and Aerobic Glycolysis as Targeting Strategy Against mpox Virus Replication

**DOI:** 10.1002/advs.202509148

**Published:** 2025-10-27

**Authors:** Pengjun Wei, Zongzheng Zhao, Ruoqi Xu, Qin Yan, Liangzi Jiang, Fuxiao Geng, Yang Gu, Tianjiao Wang, Jing Zhou, Xiao Li, Qin Yan, Chun Lu, Wan Li

**Affiliations:** ^1^ Department of Microbiology Nanjing Medical University Nanjing 211166 P. R. China; ^2^ Changchun Veterinary Research Institute Chinese Academy of Agricultural Sciences Changchun 130122 P. R. China; ^3^ Key Laboratory for Pathogen Infection and Control of Jiangsu Province Nanjing Medical University Nanjing 211166 P. R. China; ^4^ Department of Infectious Diseases Changzhou Third People's Hospital Changzhou Medical Center Nanjing Medical University Nanjing 211166 P. R. China

**Keywords:** aerobic glycolysis, crotonylation, I3, mpox virus, MYST1

## Abstract

The global outbreak of mpox caused by the mpox virus (MPXV) in 2022 and 2024 underscores the urgent need to elucidate mechanisms governing viral replication during pathogenesis. Metabolic reprogramming is a conserved hallmark of viral infections, however, the precise mechanisms by which MPXV manipulates host cell metabolism remain unknown. Here, it is demonstrated that MPXV hijacks aerobic glycolysis via lysine crotonylation of its I3 protein, which is essential for MPXV replication. Mechanistically, MYST histone acetyltransferase 1 (MYST1), an acetyltransferase upregulated by MPXV, binds to and catalyzes the crotonylation of I3. The crotonylated I3 interacts with WD‐repeat protein 26 (WDR26) to prevent its ubiquitination‐dependent degradation, leading to enhanced aerobic glycolysis and promoting MPXV replication. Either pharmacological inhibition of MYST1 using MC4033 or blocking aerobic glycolysis with the glycolytic inhibitors 2‐Deoxy‐D‐glucose (2‐DG) or dichloroacetic acid (DCA) effectively suppresses MPXV replication. These findings uncover a novel crotonylation‐dependent mechanism through which MPXV reprograms host metabolism to facilitate viral propagation, and identify lysine crotonylation and aerobic glycolysis as potential therapeutic targets against mpox.

## Graphical abstract

Wei et al. report that MPXV infection induces aerobic glycolysis, a process mediated by the viral protein I3 through lysine crotonylation at its K102 residue. The acetyltransferase MYST1 catalyzes the crotonylation of I3 to inhibit the ubiquitin‐mediated degradation of WDR26. Consequently, this process promotes both aerobic glycolysis and viral replication. Targeting either MYST1 or aerobic glycolysis significantly impairs MPXV replication.

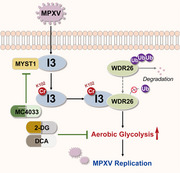

## 1 Introduction

The mpox virus (MPXV) is the causative agent of mpox, a zoonotic infectious disease marked by rash, lymphadenopathy, and fever.^[^ ^1^ ^]^ Initially identified in monkeys in 1958,^[^ ^2^ ^]^ MPXV later emerged in humans, with the first case reported in 1970 in the Democratic Republic of the Congo, subsequently establishing endemicity in West and Central Africa.^[^ ^3^ ^]^ Due to the global outbreak in 2022 and 2024, the World Health Organization (WHO) declared mpox as a Public Health Emergency of International Concerns.^[^ ^4^ ^]^ As of January 2025, MPXV had been reported in 130 countries and regions, with over 129537 confirmed cases and 283 fatalities.^[^ ^5^ ^]^ Current preventive and therapeutic strategies against MPXV exhibit significant limitations. While encouraging advancements have been achieved in recent studies on MPXV vaccines, current vaccines offer only partial protection against MPXV infection,^[^ ^6^, ^7^, ^8^, ^9^ ^]^ and other agents such as cidofovir, brincidofovir, and tecovirimat have shown unsatisfactory outcomes.^[^ ^6^, ^10^, ^11^, ^12^ ^]^ Therefore, a renewed understanding of MPXV replication is imperative for the design of more specific and effective antiviral therapeutics.

MPXV, a member of the orthopoxvirus genus, possesses a double‐stranded DNA genome with brick‐shaped or oval virions measuring ≈200–250 nm. Its linear genome, ≈200 kb in length, contains more than 190 open reading frames (ORFs) and exhibits a characteristic structure shared among orthopoxviruses: a highly conserved central core region flanked by variable terminal regions and inverted terminal repeats.^[^ ^13^ ^]^ Over 90% of sequences in the central core share homology with other orthopoxviruses.^[^ ^14^ ^]^ Employing its encoded multi‐subunit DNA‐dependent RNA polymerase (RNAP), MPXV is exclusively replicated in the cytoplasm,^[^ ^15^ ^]^ and proliferates into two infectious enveloped virions: extracellular enveloped virus (EEV) and intracellular mature virus (IMV).^[^ ^6^, ^16^ ^]^ Nonetheless, the molecular mechanisms underlying MPXV replication should be further clarified.

Viruses, lacking an autonomous metabolic network, have developed diverse strategies for rewiring the metabolic system of their host to hijack the host's metabolic resources for replication. Aerobic glycolysis, commonly referred to as the Warburg effect, was initially observed in cancer cells.^[^ ^17^ ^]^ These cells exhibit elevated glucose uptake and predominantly metabolize glucose through pyruvate to lactate via lactate dehydrogenase (LDH), rather than utilizing it for ATP production through mitochondrial respiration and oxidative phosphorylation (OXPHOS).^[^ ^18^ ^]^ Accumulating evidence suggests that the majority of viral infections exploit aerobic glycolysis to fulfill the heightened demands for virion production. For instance, SARS‐CoV‐2 infection triggers mitochondrial reactive oxygen species (ROS) production, which induces stabilization of hypoxia‐inducible factor‐1α (HIF‐1α) and promotes aerobic glycolysis by enhancing the transcription of glycolytic genes, ultimately facilitating SARS‐CoV‐2 replication.^[^ ^19^ ^]^ Aerobic glycolysis supports hepatitis B virus (HBV) protein synthesis through interaction between viral surface antigen and pyruvate kinase isoform M2 (PKM2), a key regulator of glucose metabolism.^[^ ^20^ ^]^ Our prior study showed that Kaposi's sarcoma‐associated herpesvirus (KSHV)‐encoded viral interferon regulatory factor 1 (vIRF1) degrades RNA‐binding protein heterogeneous nuclear ribonuclear protein Q1 to enhance aerobic glycolysis via recruiting E3 ubiquitin ligase Kelch‐Like 3 and decaying glycerophosphodiester phosphodiesterase domain containing 1 mRNA.^[^ ^21^ ^]^ Despite these findings, the role that aerobic glycolysis plays in MPXV infection and replication remains unexplored.

Protein lysine acylation, a prominent subtype of post‐translational modifications (PTMs), plays a pivotal role in regulating diverse protein functions.^[^ ^22^ ^]^ Lysine acetylation is the first identified and most extensively studied form of acylation modification. With recent advancements in mass spectrometry technology, additional lysine acylation modification types have been discovered, such as succinylation, lactylation, crotonylation, 2‐hydroxyisobutyrylation, β‐hydroxybutyrylation, and malonylation.^[^ ^23^, ^24^, ^25^, ^26^ ^]^ Lysine crotonylation (Kcr) is a newly discovered PTM, initially detected on histones in 2011.^[^ ^26^ ^]^ Subsequently, an increasing number of non‐histone proteins have been found to be crotonylated, and these proteins are involved in diverse biological processes, including myocardial injury, tumorigenesis, kidney fibrosis, and cardiac hypertrophy.^[^ ^27^, ^28^, ^29^ ^]^ This modification is dynamically regulated by specific “writer” or “eraser” enzymes that respectively add or remove crotonyl groups from lysine residues. The regulators of crotonylation include acetyltransferases like E1A‐binding protein p300/CREB‐binding protein,^[^ ^30^ ^]^ MYST (MOZ, Ybf2/Sas3, Sas2, and Tip60) family proteins,^[^ ^31^ ^]^ general control non‐depressible 5 ^[^ ^32^ ^]^ and lysine acetyltransferase 7,^[^ ^33^ ^]^ as well as deacyltransferases such as histone deacetylases (HDACs) ^[^ ^34^ ^]^ and sirtuins.^[^ ^35^ ^]^ A study showed that acetyl‐CoA synthetase 2 mediates histone crotonylation to promote HIV reactivation.^[^ ^36^ ^]^ However, the role of crotonylation in MPXV infection remains unclear.

Here, we found that MPXV infection triggers aerobic glycolysis through crotonylation of the viral I3 protein at lysine 102. Mechanistically, the acetyltransferase MYST1 facilitates the crotonylation of I3, thereby enhancing the interaction between I3 and WDR26. This interaction diminishes the ubiquitination‐mediated degradation of WDR26. Notably, targeting MYST1 or aerobic glycolysis effectively suppresses MPXV replication. These results indicate that the crotonylation of viral proteins exploits host metabolic pathways to promote MPXV replication, a mechanism that holds promise for the development of targeted therapeutic interventions.

## 2 Results

### 2.1 Mpox Virus Infection Promotes Aerobic Glycolysis Through Its‐Encoded Protein I3

**Figure 1:**
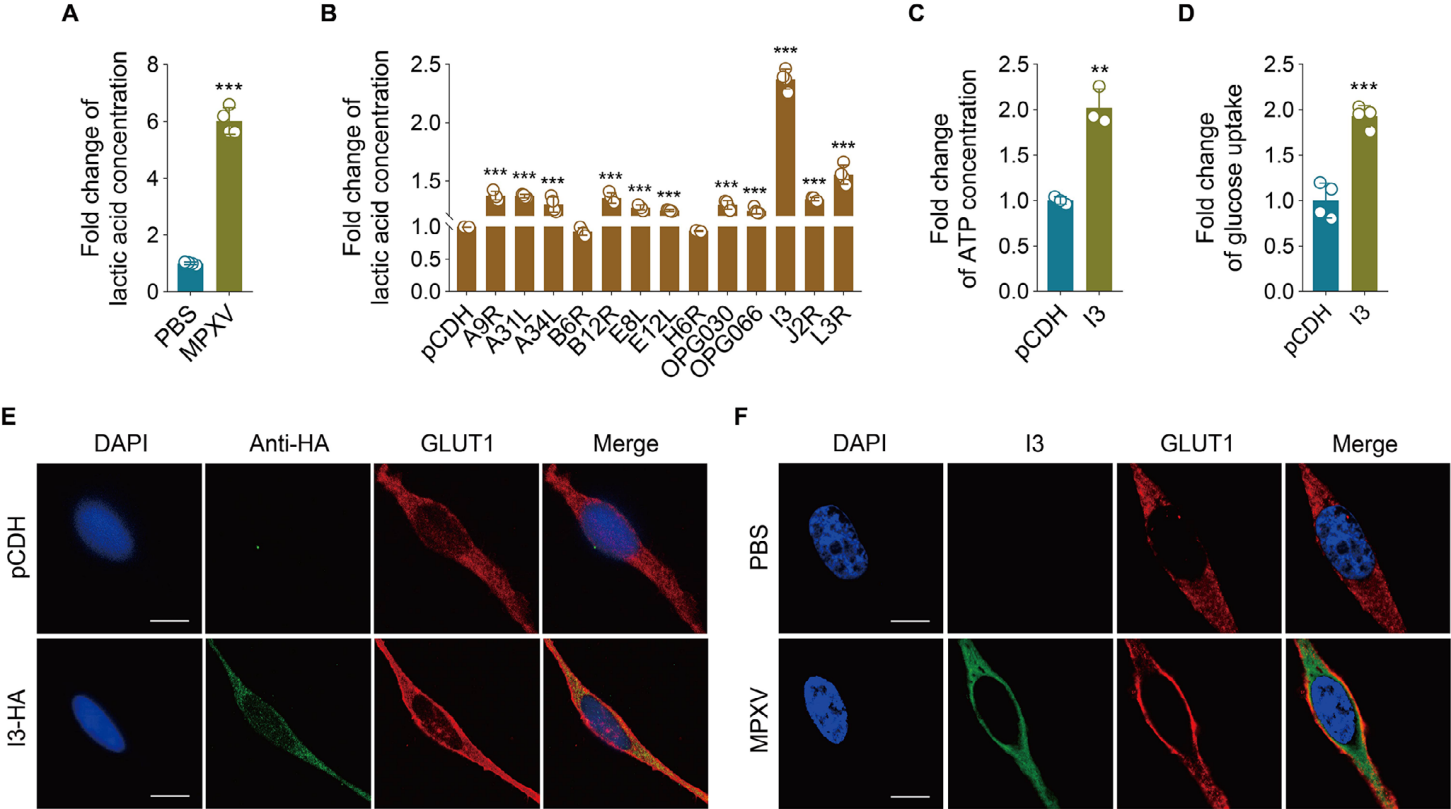
Mpox virus infection promotes aerobic glycolysis through its encoded protein I3. A) Lactate production was detected in HeLa cells treated with PBS (**PBS**) or infected with MPXV (**MPXV**) for 24 h (*n* = 4). B) Lactate production was examined in HeLa cells that were transduced to express 13 MPXV genes (A9R, A31L, A34L, B6R, B12R, E8L, E12L, H6R, OPG030, OPG066, I3, J2R, L3R), respectively (*n* = 4). C) The level of intracellular ATP in HeLa cells infected with lentiviral I3 (**I3‐HA**) or control lentivirus (**pCDH**) (*n* = 3). D) Glucose uptake was measured in cells treated as in (**C**) (*n* = 4). E) The intracellular localization of GLUT1 in HeLa cells treated as in (**C**) was detected by immunofluorescence staining. The scale bar was 10 µm. F) The intracellular localization of GLUT1 in HeLa cells treated with PBS (**PBS**) or infected with MPXV (**MPXV**) was detected by immunofluorescence staining. The protein I3 was utilized as an indicator for confirmation of MPXV infection. The scale bar was 10 µm. Data are shown as mean ± SD. ^**^ *p* < 0.01 and ^***^ *p* < 0.001, Student's *t*‐test.

To explore the relationship between aerobic glycolysis and MPXV infection, we employed HeLa cells, a validated cell model for orthopoxvirus studies.^[^ ^37^, ^38^ ^]^ Lactate, as the terminal product, is a pivotal metabolic indicator of aerobic glycolysis. Our previous study revealed a marked increase in lactate production in THP‐1 cells infected with MPXV.^[^ ^39^ ^]^ Consistently, MPXV‐infected HeLa cells exhibited a pronounced increase in lactate production (**Figure** **1**A), indicating that MPXV infection stimulates aerobic glycolysis. To identify viral effectors driving this glycolytic reprogramming, we screened 13 MPXV genes (A9R, A31L, A34L, B6R, B12R, E8L, E12L, H6R, OPG030, OPG066, I3, J2R, L3R). These genes were specifically chosen on the basis of sequence conservation and their predicted relevance to viral replication.^[^ ^13^ ^]^ Lentivirus‐mediated expression revealed that I3 expression induced the highest levels of lactate production (Figure 1B). Furthermore, I3 expression significantly elevated intracellular ATP levels and glucose uptake, two additional hallmarks of aerobic glycolysis (Figure 1C,D). Since glycolysis depends on the movement of glucose transporters (GLUTs) to the plasma membrane,^[^ ^40^ ^]^ we assessed GLUT1 and GLUT3 distribution. I3 expression specifically promoted GLUT1 translocation to the plasma membrane (Figure 1E; Figure S1A, Supporting Information), while GLUT3 localization remained unaltered (Figure S1B, Supporting Information). The expression of either H6R or B6R did not exert any influence on the translocation of GLUT1 (Figure S1C, Supporting Information). Strikingly, MPXV infection also induced the translocation of GLUT1 to the membrane (Figure 1F; Figure S1D, Supporting Information). Collectively, these results demonstrate that MPXV infection enhances aerobic glycolysis, with viral I3 being a major contributor.

### 2.2 Crotonylation of I3 at Lysine 102 Drives I3‐Regulated Aerobic Glycolysis

**Figure 2:**
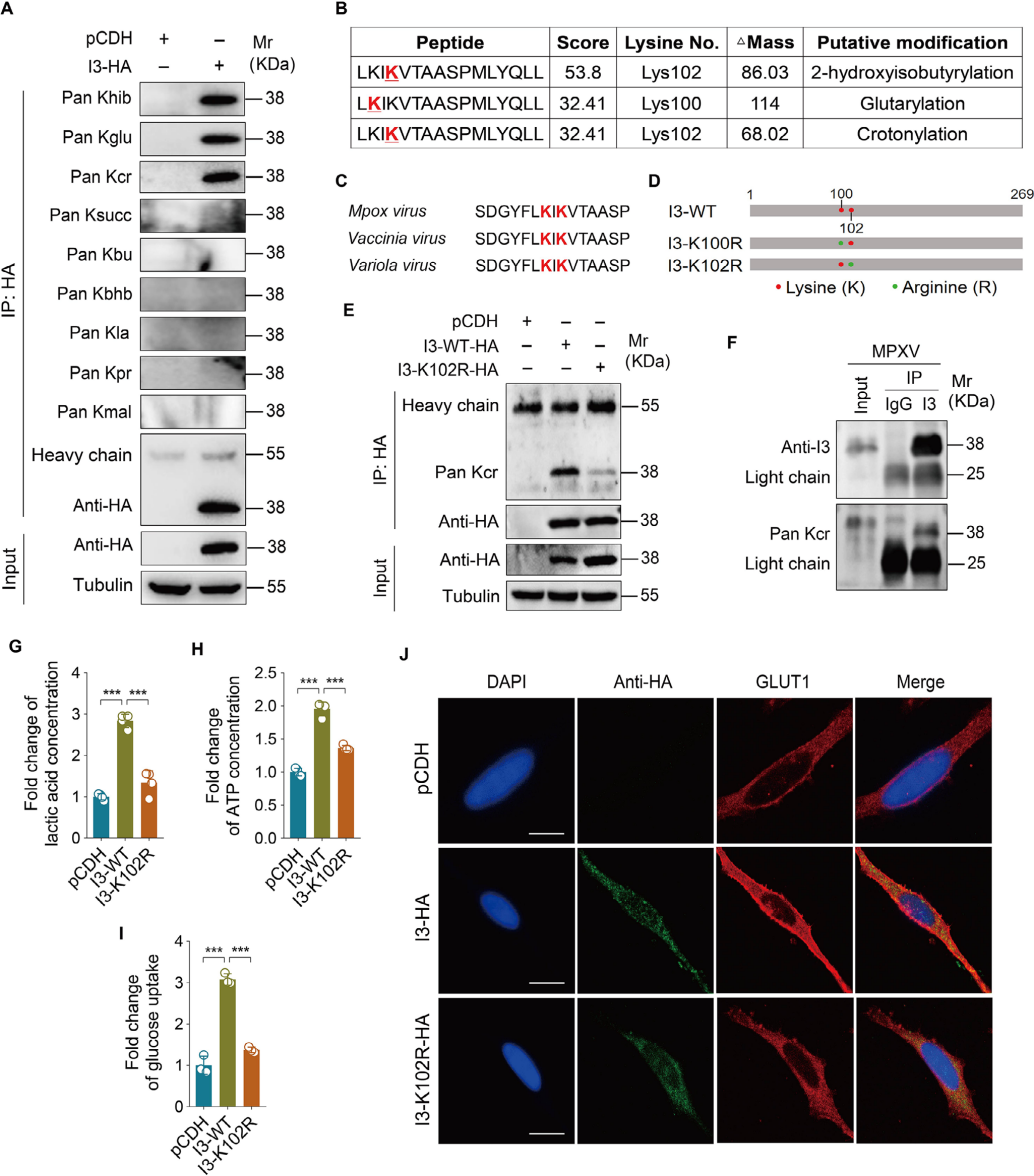
Crotonylation of I3 at lysine 102 drives I3‐regulated aerobic glycolysis. A) HeLa cells were transduced with lentiviral I3 (**I3‐HA**) or control lentivirus (**pCDH**). Following transduction, cell lysates were subjected to immunoprecipitation using an anti‐HA antibody. Immunoprecipitates were then analyzed by immunoblotting with antibodies specific for distinct lysine modifications, including an anti‐Khib antibody, an anti‐Kglu antibody, an anti‐Kcr antibody, an anti‐Ksucc antibody, an anti‐Kbu antibody, an anti‐Kbhb antibody, an anti‐Kla antibody, an anti‐Kpr antibody, and an anti‐Kmal antibody. B) HeLa cells transduced with lentiviral vectors expressing I3 (**I3‐HA**) or control lentivirus (**pCDH**) were subjected to immunoprecipitation, followed by SDS‐PAGE and liquid chromatography‐tandem mass spectrometry (LC‐MS/MS) analysis. The resulting mass spectrometry data included the amino acid sequence of identified peptides, the match score of the peptide, and the specific lysine (K) residue positions. Observed lysine mass shifts of +86.03, +114.04, and +68.02 Da were attributed to 2‐hydroxybutyrylation, glutarylation, and crotonylation modifications, respectively. C) The sequences surrounding I3 K100 and K102 in different orthopoxvirus genus viruses were mapped, with conserved lysine residues marked in red. D) Schematic representation of I3 with the indicated locations of mutated lysine (K) sites. The lysine site of wild‐type I3 (**I3‐WT**) was marked in red, and the site mutated to arginine was marked in green. The lysine 100 mutation was designated as I3‐K100R. The lysine 102 mutation was designated I3‐K102R. E) HeLa cells were transduced with wild‐type I3 (**I3‐WT‐HA**) or I3 mutant (**I3‐K102R‐HA**), respectively. An IP assay was performed to examine the level of I3 crotonoylation. F) HeLa cells infected with MPXV were pulled down by anti‐IgG antibody or anti‐I3 antibody and detected by western blotting with anti‐I3 antibody and anti‐Kcr antibody. G) Cells treated as in (**E**) were used to measure the level of lactate production (*n* = 4). H) Cells treated as in (**E**) were used to measure the level of intracellular ATP (*n* = 4). I) Cells treated as in (**E**) were used to measure the level of glucose uptake (*n* = 3). J) HeLa cells treated as in (**E**) were used to measure the localization of GLUT1 by immunofluorescence staining. The scale bar was 10 µm. Data are shown as mean ± SD. ^***^ *p* < 0.001, Student's *t*‐test.

To explore potential lysine acylation modifications on I3, we conducted immunoprecipitation screens encompassing nine types of lysine acylations: 2‐hydroxyisobutyrylation (Khib), glutarylation (Kglu), crotonylation (Kcr), succinylation (Ksucc), butyrylation (Kbu), β‐hydroxybutyrylation (Kbhb), lactylation (Kla), propionylation (Kpr), and malonylation (Kmal). The results revealed that I3 protein likely undergoes 2‐hydroxyisobutyrylation, glutarylation, and crotonylation (**Figure** **2**A). Subsequent liquid chromatography‐mass spectrometry analysis pinpointed that lysine 102 (K102) of I3 is a probable site for both 2‐hydroxyisobutyrylation and crotonylation, while K100 emerged as a potential glutarylation site (Figure 2B; Figure S2, Supporting Information). Conservation analysis revealed high conservation of K100 and K102 across orthopoxviruses (Figure 2C). Then, we generated lysine‐to‐arginine(R) mutations of K100 and K102 (K100R and K102R) (Figure 2D), to mimic a deacylated state.^[^ ^41^, ^42^, ^43^ ^]^ The K102R mutation reduced I3 crotonylation (Figure 2E), but did not affect 2‐hydroxyisobutyrylation (Figure S3A, Supporting Information), whereas the K100R mutation had no impact on glutarylation (Figure S3B, Supporting Information). Immunoprecipitation with anti‐I3 antibody further confirmed that endogenous I3 experienced crotonylation during MPXV infection (Figure 2F). These results demonstrated that MPXV‐encoded I3 undergoes crotonylation and K102 is its major crotonylation site.

To determine whether I3 crotonylation is involved in I3‐mediated aerobic glycolysis, we ectopically expressed wild‐type I3 and the K102R mutant in HeLa cells and assessed lactate production, ATP production, and glucose uptake. Our results indicated that the absence of crotonylation at K102 reduced the ability of I3 to enhance lactate production, ATP generation, and glucose uptake (Figure 2G–I). Furthermore, the K102R mutant was unable to facilitate GLUT1 translocation to the membrane (Figure 2J; Figure S4, Supporting Information).

These data underscore the essentiality of crotonylation at K102 of I3 for its regulatory role in aerobic glycolysis.

### 2.3 MYST1 Catalyzes Crotonylation of I3 to Enhance Aerobic Glycolysis

**Figure 3:**
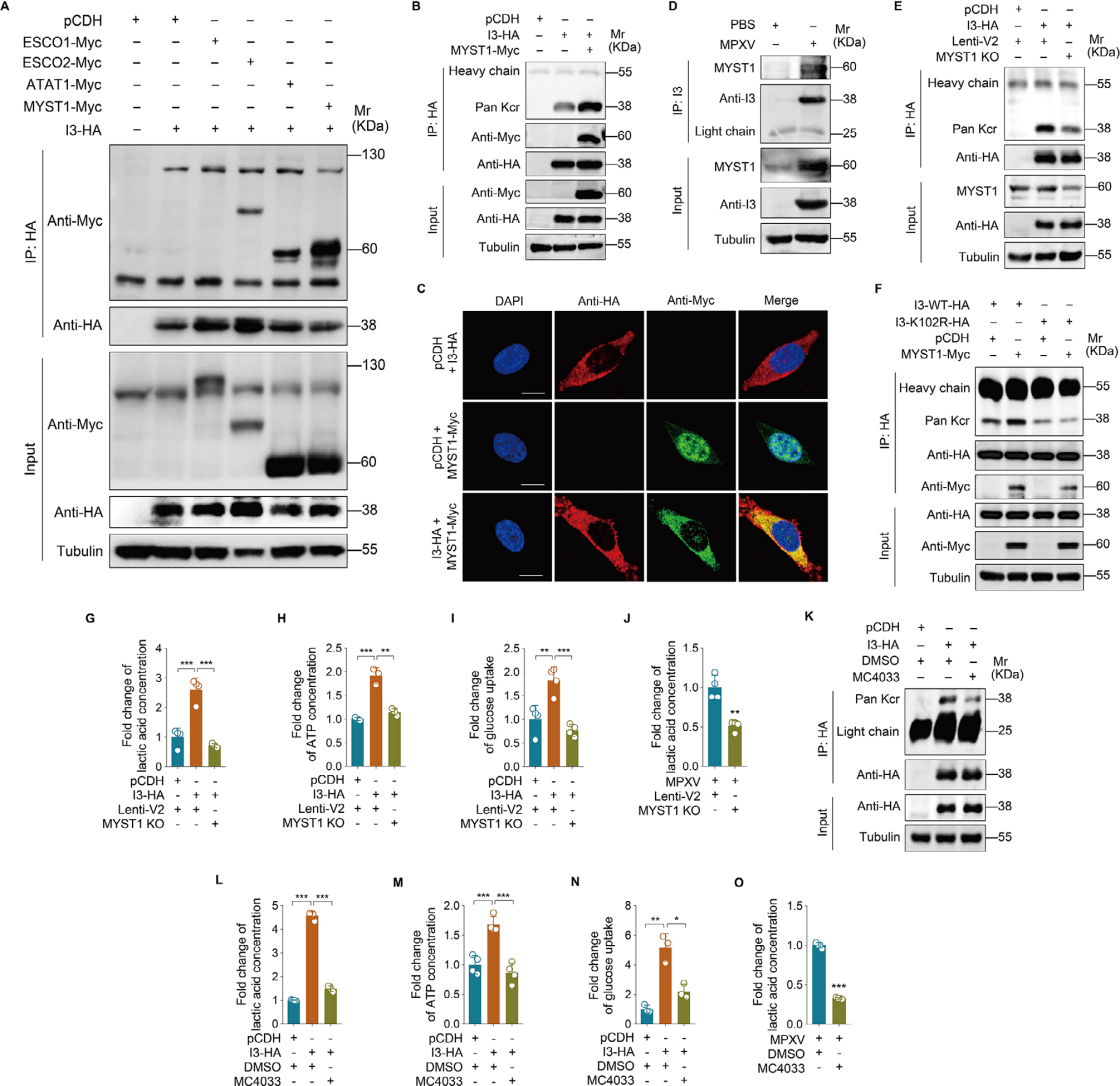
MYST1 catalyzes crotonylation of I3 to enhance aerobic glycolysis. A) HeLa cells transduced with I3 (**I3‐HA**) or its control (**pCDH**) were subsequently infected with lentiviral acyltransferases ESCO1, ESCO2, MYST1, and ATAT1 (**ESCO1‐Myc**, **ESCO2‐Myc**, **MYST1‐Myc,** and **ATAT1‐Myc**). An IP assay was conducted using an anti‐HA antibody to identify acyltransferases that interact with I3. B) HeLa cells transduced with I3 (**I3‐HA**) or its control (**pCDH**) were infected with MYST1(**MYST1‐Myc**) or its control (**pCDH**). An IP assay was conducted using an anti‐HA antibody to examine the crotonylation level of I3. C) HeLa cells co‐transduced with I3 (**I3‐HA**) and MYST1 (**MYST1‐Myc**) were used to measure the intracellular localization of I3 and MYST1 by immunofluorescence staining. The scale bar was 10 µm. D) HeLa cells were infected with MPXV. An IP assay was conducted using an anti‐I3 antibody to examine the interaction between I3 and MYST1. E) MYST1 knockout (**MYST1 KO**) HeLa cells were infected with I3 (**I3‐HA**) or its control (**pCDH**). An IP assay was conducted using an anti‐HA antibody to examine the crotonylation level of I3. F) HeLa cells infected with wild‐type I3 (**I3‐WT‐HA**) or I3 mutant (**I3‐K102R‐HA**), were transduced with MYST1(**MYST1‐Myc**) or its control (**pCDH**). An IP assay was conducted using an anti‐HA antibody to examine the crotonylation level of I3. G) Cells treated as in (**E**) were used to measure the level of lactate production (*n* = 4). H) Cells treated as in (**E**) were used to measure the level of intracellular ATP (*n* = 3). I) Cells treated as in (**E**) were used to measure the level of glucose uptake (*n* = 4). J) MYST1 knockout (**MYST1 KO**) HeLa cells infected with MPXV were used to measure the level of lactate production (*n* = 4). K) HeLa cells transduced with I3 (**I3‐HA**) or its control (**pCDH**) were treated with the MYST1 inhibitor MC4033 (50 µm) for 48 h. An IP assay was conducted using an anti‐HA antibody to examine the crotonylation level of I3. L) Cells treated as in (**K**) were used to measure the level of lactate production (*n* = 4). M) Cells treated as in (**K**) were used to measure the level of intracellular ATP (*n* = 4). N) Cells treated as in (**K**) were used to measure the level of glucose uptake (*n* = 3). O) HeLa cells were infected with MPXV for 2 h, followed by treating with the MYST1 inhibitor MC4033 at a concentration of 50 µm for 48 h, and subsequently assayed to determine the level of lactate production (*n* = 4). Data are shown as mean ± SD. ^*^ *p* < 0.05, ^**^ *p* < 0.01 and ^***^ *p* < 0.001, Student's *t*‐test.

To identify the acyltransferase responsible for I3 crotonylation, we conducted a screen of four candidate acetyltransferases (ESCO1, ESCO2, ATAT1, and MYST1) by co‐expressing them with I3. Co‐immunoprecipitation assays revealed the interactions between I3 and ESCO2, ATAT1, and MYST1 (**Figure** **3**A); however, only MYST1 overexpression robustly increased I3 crotonylation (Figure 3B; Figure S5, Supporting Information). Both immunofluorescence and nuclear‐cytoplasmic fractionation analyses showed that I3 expression induced the translocation of MYST1 from the nucleus to the cytoplasm (Figure 3C; Figure S6A,B, Supporting Information). The interaction between I3 and MYST1 was further corroborated by endogenous MYST1 co‐immunoprecipitating with I3 during MPXV infection (Figure 3D). MYST1 expression was markedly upregulated upon MPXV infection (Figure 3D; Figure S6C, Supporting Information). Meanwhile, MPXV infection also induced the translocation of MYST1 from the nucleus to the cytoplasm (Figure S6D,E, Supporting Information). CRISPR‐Cas9‐mediated MYST1 knockout (KO) in HeLa cells reduced I3 crotonylation (Figure 3E; Figure S6F, Supporting Information), further confirming the regulation of MYST1 on I3 crotonylation. The co‐expression analysis of I3‐K102R and MYST1 revealed no discernible change in the level of I3 crotonylation (Figure 3F), indicating that MYST1 specifically crotonylates I3 at K102.

We next investigated MYST1's involvement in aerobic glycolysis regulation. MYST1 knockout suppressed I3‐mediated increases in lactate production, ATP generation, and glucose uptake (Figure 3G–I). Similarly, MPXV‐infected MYST1 KO cells exhibited a significant reduction in lactate production (Figure 3J). To further substantiate these findings, we employed MC4033, a specific MYST1 inhibitor. Treatment with MC4033 suppressed I3 crotonylation (Figure 3K), and impaired aerobic glycolysis, as shown by reduced lactate production, ATP production, and glucose uptake (Figure 3L–N). This inhibitory effect was also observed in MPXV‐infected cells treated with MC4033 (Figure 3O). Collectively, these findings demonstrate that MPXV infection‐upregulated MYST1 catalyzes the crotonylation of I3 to enhance aerobic glycolysis.

### 2.4 Crotonylation of I3 Enhances Aerobic Glycolysis by Inhibiting WDR26 Degradation

**Figure 4:**
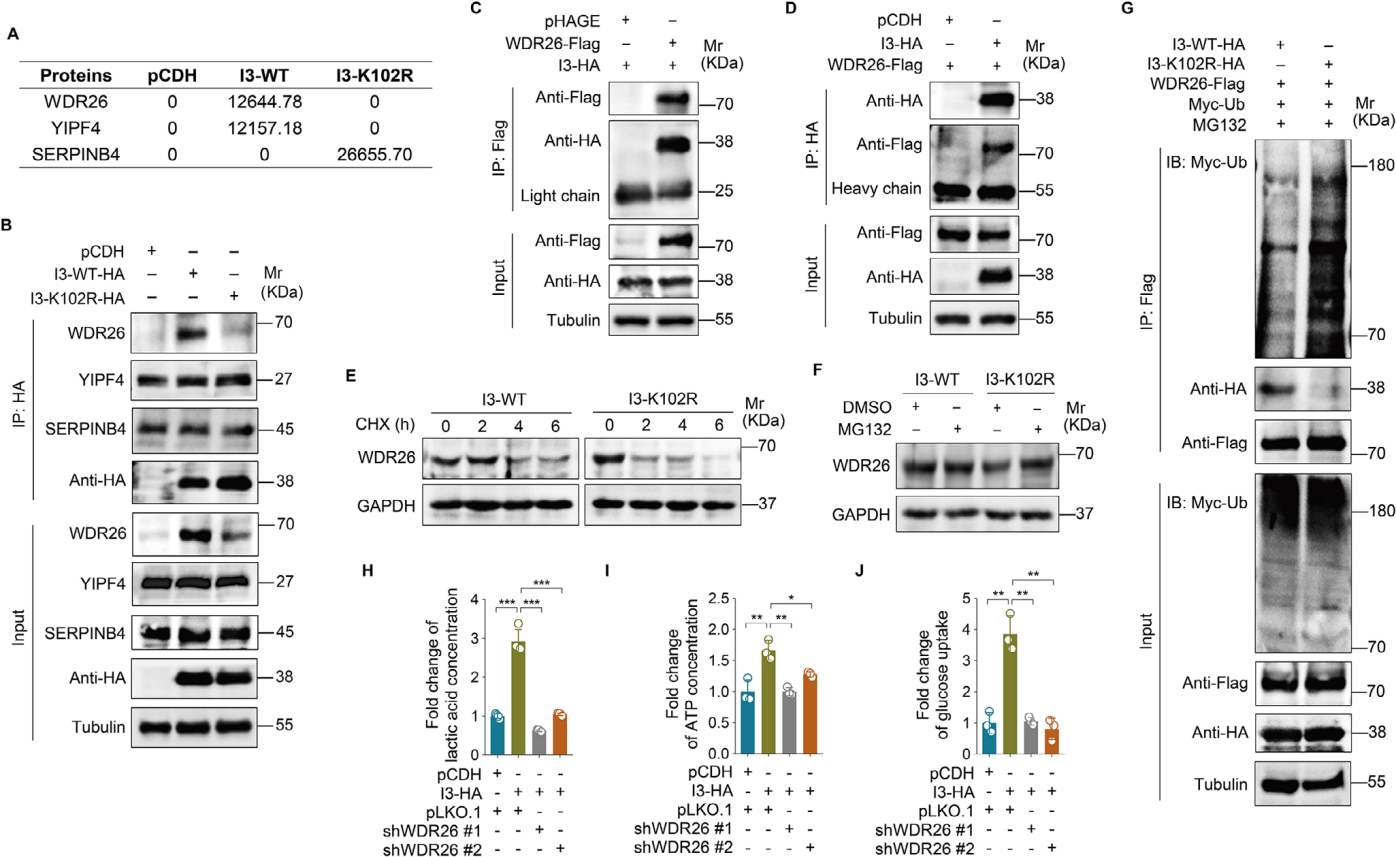
Crotonylation of I3 enhances aerobic glycolysis by inhibiting WDR26 degradation. A) Mass spectrometry analysis of the differential binding proteins in wild‐type I3 (**I3‐WT‐HA**)‐ and I3 mutant‐expressing HeLa cells (**I3‐K102R‐HA**). B) HeLa cells were transduced with wild‐type I3 (**I3‐WT‐HA**) or I3 mutant (**I3‐K102R‐HA**). An IP assay was performed to assess the interaction between I3‐WT or I3‐K102R and candidate proteins (WDR26, YIPF4, and SERPINB4). C) I3‐expressed HeLa cells (**I3‐HA**) were infected with lentiviral WDR26‐Flag (**WDR26‐Flag**) or its control **(pCDH**). An IP assay was conducted using an anti‐Flag antibody to examine the interaction between I3 and WDR26. D) WDR26‐overexpressing HeLa cells (**WDR26‐Flag**) were infected with lentiviral I3‐HA (**I3‐HA**) or its control (**pCDH**). An IP assay was conducted using an anti‐HA antibody to examine the interaction between WDR26 and I3. E) HeLa cells transduced with wild‐type I3 (**I3‐WT‐HA**) or I3 mutant (**I3‐K102R‐HA**) were treated with CHX (10 µg mL^−1^) for 0, 2, 4, and 6 h. Western blotting analysis was used to detect the level of WDR26 expression. F) HeLa cells transduced with wild‐type I3 (**I3‐WT‐HA**) or I3 mutant (**I3‐K102R‐HA**) were treated with MG132 (10 µm) for 2 h. Western blotting analysis was used to detect the level of WDR26 expression. G) HeLa cells transduced with either wild‐type I3 (**I3‐WT‐HA**) or I3 mutant (**I3‐K102R‐HA**) were subsequently transfected with plasmids encoding Myc‐tagged ubiquitin (**Myc‐Ub**) and Flag‐tagged WDR26 (**WDR26‐Flag**). Following transfection, cells were treated with 10 µm MG132 for 2 h. The ubiquitination status of WDR26 was assessed by immunoprecipitation using anti‐Flag antibody. H) HeLa cells transduced with shWDR26 (**shWDR26 #1** and **shWDR26 #2**) or its control (**pLKO.1**) were used to measure the level of lactate production (*n* = 4). I) Cells treated as in **(H)** were used to measure the level of intracellular ATP (*n* = 3). J) Cells treated as in (**H**) were used to measure the level of glucose uptake (*n* = 3). Data are shown as mean ± SD. ^*^ *p* < 0.05, ^**^ *p* < 0.01, and ^***^ *p* < 0.001, Student's *t*‐test. *n.s*., not significant.

To identify proteins that interact differentially with I3‐WT and I3‐K102R, we conducted mass spectrometry analysis and selected the top three candidates for further investigation (**Figure** **4**A). Co‐immunoprecipitation revealed a specific interaction between I3 and WDR26, and this interaction is weaker in the decrotonylated I3‐K102R mutant (Figure 4B). Exogenous co‐immunoprecipitation in HeLa cells co‐expressing I3‐WT and WDR26 confirmed this interaction (Figure 4C,D). Notably, the expression of I3‐WT, but not I3‐K102R, led to an increase in WDR26 protein levels without altering WDR26 mRNA levels (Figure 4B; Figure S7, Supporting Information). A significant increase in WDR26 protein levels was also observed in MPXV‐infected cells (Figure S8, Supporting Information). To investigate the effect of I3 crotonylation on WDR26 stability, we treated cells expressing either I3‐WT or I3‐K102R with cycloheximide (CHX), a known inhibitor of protein synthesis. I3‐K102R facilitated WDR26 degradation compared to I3‐WT (Figure 4E; Figure S9A, Supporting Information). Treatment with MG132, a proteasome inhibitor, blocked I3‐K102R‐induced WDR26 degradation (Figure 4F; Figure S9B, Supporting Information). Furthermore, I3‐K102R promoted ubiquitination‐dependent degradation of WDR26 (Figure 4G). However, when we expressed I3‐WT and I3‐K102R in MYST1 KO cells, we found that neither I3‐WT nor I3‐K102R interacted with WDR26 (Figure S10A, Supporting Information). Moreover, there were no significant differences between I3‐WT and I3‐K102R in their effects on WDR26 expression, or WDR26 degradation and ubiquitination (Figure S10B–F, Supporting Information).

To gain further insights into the role of WDR26 in aerobic glycolysis, we knocked down WDR26 using short hairpin RNAs (shRNAs) in I3‐expressing HeLa cells (Figure S11, Supporting Information). WDR26 depletion suppressed the I3‐induced increases in lactate production, ATP generation, and glucose uptake (Figure 4H–J). Together, these data suggest that the crotonylation of I3 enables its interaction with WDR26, thereby protecting WDR26 from ubiquitination‐dependent degradation and ultimately promotes aerobic glycolysis.

### 2.5 Targeting MYST1 or Aerobic Glycolysis Inhibits MPXV Replication

**Figure 5:**
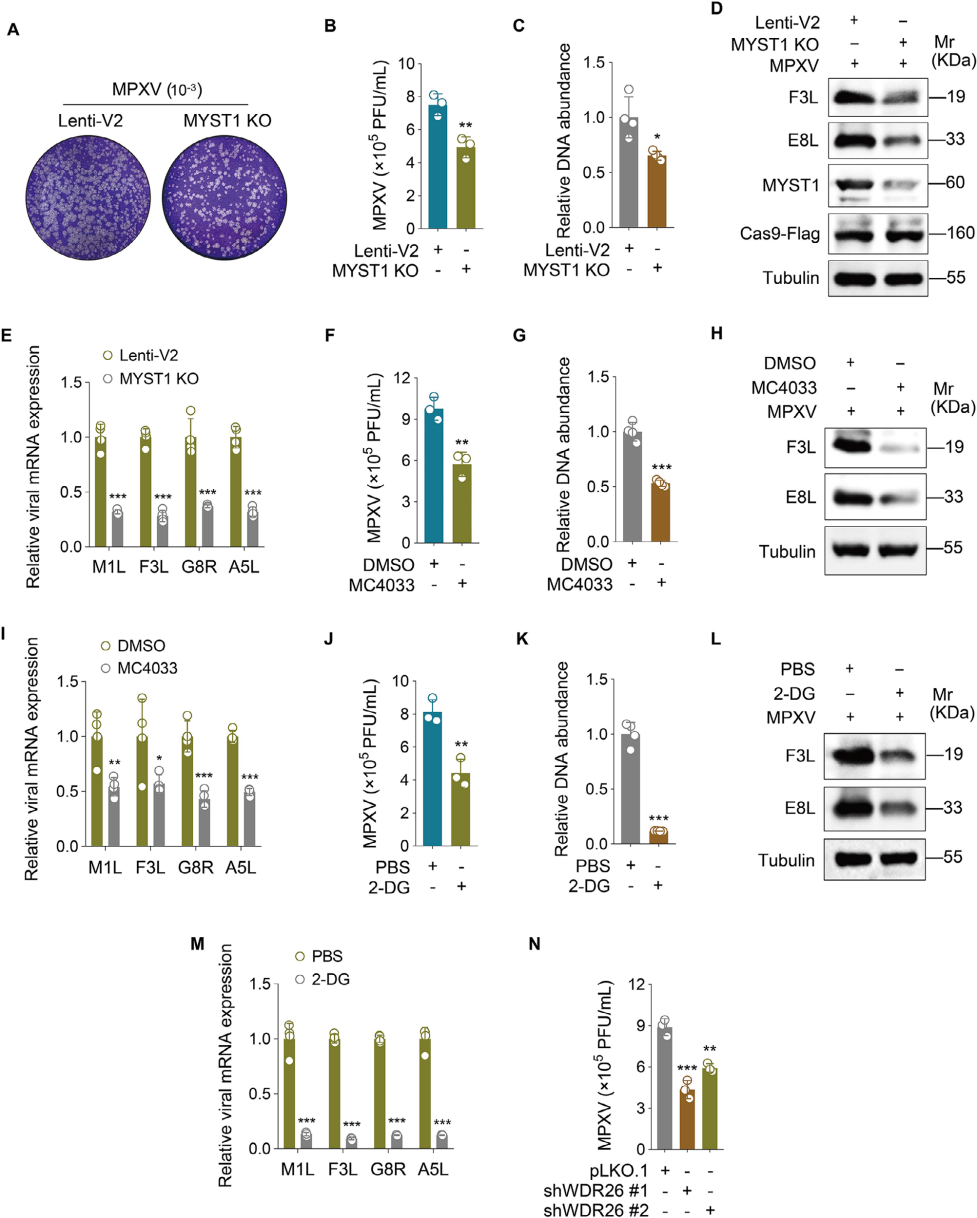
Targeting MYST1 or aerobic glycolysis inhibits MPXV replication. A) MYST1 knockout (**MTSY1 KO**) HeLa cells were infected with MPXV and used for conducting a plaque assay. The medium was replaced 1 h after MPXV infection, and the infected cells continued to be cultured for another 24 h before the plaque assay. Representative images of plaques were taken from randomly selected areas in each sample. B) Quantification of the results in (**A**) (*n* = 3). C) MYST1 knockout (**MYST1 KO**) HeLa cells were infected with MPXV for 24 h. Viral genome copies were detected by viral genome quantification assay (*n* = 4). D) Cells treated as in (**C**) were used to measure the levels of MPXV proteins (F3L and E8L). E) Cells treated as in (**C**) were used to measure the mRNA levels of MPXV genes (M1L, F3L, G8R, and A5L) (*n* = 4). F) HeLa cells were infected with MPXV for 2 h, followed by treating with the MYST1 inhibitor MC4033 (50 µm) for 48 h, and used for conducting a plaque assay (*n* = 3). G) HeLa cells were infected with MPXV for 2 h followed by treating with the MYST1 inhibitor MC4033 (50 µm) for 48 h. Viral genome copies were detected by viral genome quantification assay (*n* = 4). H) Cells treated as in (**G**) were used to measure the protein levels of MPXV genes (F3L and E8L). I) Cells treated as in (**G**) were used to measure the mRNA level of MPXV genes (M1L, F3L, G8R, and A5L) (*n* = 4). J) HeLa cells were infected with MPXV for 2 h, followed by treating with the glycolysis inhibitor 2‐DG (5 mm) for 48 h, and used for conducting a plaque assay (*n* = 3). K) HeLa cells were infected with MPXV for 2 h, followed by treating with the glycolysis inhibitor 2‐DG (5 mm) for 48 h. Viral genome copies were detected by viral genome quantification assay (*n* = 4). L) Cells treated as in (**K**) were used to measure the protein levels of MPXV genes (F3L and E8L). M) Cells treated as in (**K**) were used to measure the mRNA level of MPXV genes (M1L, F3L, G8R, and A5L) (*n* = 4). N) HeLa cells were transduced with either shWDR26 (**shWDR26 #1** and **shWDR26 #2**) or its control (**pLKO.1**), then infected with MPXV, and subsequently subjected to plaque assay (*n* = 3). Data are shown as mean ± SD. ^*^ *p* < 0.05, ^**^ *p* < 0.01 and ^***^ *p* < 0.001, Student's *t*‐test.

To determine whether MYST1‐mediated I3 crotonylation and its induction of aerobic glycolysis influence MPXV replication, we first evaluated MYST1's contribution. A plaque assay revealed reduced viral titers in MYST1‐depleted cells (**Figure** **5**A,B). Additionally, MYST1 depletion led to a reduction of viral genome copies in infected cells (Figure 5C). Consistently, lacking MYST1 diminished the expression of viral proteins and transcripts (Figure 5D,E). In agreement with these observations, pharmacological inhibition of MYST1 with MC4033 impaired MPXV replication (Figure 5F–I). To directly link aerobic glycolysis to viral propagation, we blocked aerobic glycolysis using 2‐deoxy‐D‐glucose (2‐DG), which markedly suppressed MPXV replication (Figure 5J–M). Moreover, 2‐DG further suppressed MPXV replication in MYST1 KO cells (Figure S12, Supporting Information). Additionally, we adopted another glycolysis inhibitor, dichloroacetic acid (DCA), which specifically inhibits pyruvate dehydrogenase kinase (PDK) and thereby suppresses aerobic glycolysis.^[^ ^44^ ^]^ Consistently, DCA treatment significantly inhibited MPXV replication (Figure S13, Supporting Information). Furthermore, WDR26 knockdown significantly attenuated viral replication (Figure 5N).

Taken together, our findings suggest that MYST1‐induced crotonylation of I3 at K102 stabilizes WDR26, thereby enhancing aerobic glycolysis and promoting MPXV replication. Suppression of MYST1 or aerobic glycolysis may represent a promising therapeutic strategy against MPXV infection.

## 3 Discussion

The spread of mpox worldwide urges breakthroughs in the research on MPXV and the development of targeted therapeutic interventions. Aerobic glycolysis, a fundamental process for viral propagation, remains unexplored in the context of MPXV. In this study, we observed that MPXV infection stimulates aerobic glycolysis. Importantly, inhibition of aerobic glycolysis with the glycolytic inhibitor 2‐DG or DCA effectively halted MPXV replication. Our findings offer novel insights into the role of aerobic glycolysis in MPXV replication and emphasize the promising therapeutic implications of targeting this metabolic pathway.

Of note, a recent study indicates that lactate, the terminal metabolite of aerobic glycolysis, impairs the innate immune response by augmenting the lactylation of cyclic GMP‐AMP synthase (cGAS).^[^ ^45^ ^]^ Based on these observations, we propose a hypothesis that, during MPXV infection, the induced aerobic glycolysis rapidly generates the energy required for viral replication while concurrently enabling lactate‐mediated immune evasion, thereby facilitating viral spread.

I3, encoded by the MPXV I3L gene, is a 34‐kDa protein that exhibits a striking sequence identity of over 98% with the I3L of Vaccinia virus (VACV), another orthopoxvirus family member.^[^ ^13^ ^]^ Previous studies on VACV have demonstrated that VACV I3 is expressed during early and intermediate phases of infection and localizes to the viral replication factories within the cytoplasm.^[^ ^46^, ^47^, ^48^ ^]^ VACV I3 functions as a critical single‐stranded DNA‐binding protein, which is essential for the viral life cycle.^[^ ^49^ ^]^ A recent investigation has unveiled an interaction between the nuclear protein FAM111A and VACV I3, promoting I3 degradation via autophagy and consequently reducing viral DNA replication.^[^ ^50^ ^]^ Despite these insights, the functional characterization of MPXV I3 remains elusive. Here, we report that overexpression of MPXV I3 results in a marked increase in lactate production, ATP generation, and glucose uptake, as well as facilitating GLUT1 translocation to the plasma membrane. These findings suggest that MPXV I3 plays a role in promoting aerobic glycolysis. Our work represents the first to delineate the function of MPXV I3, thereby broadening the functional understanding of the I3 protein within the orthopoxvirus family.

Lysine crotonylation, first identified in 2011, occurs on both histone and non‐histone proteins.^[^ ^26^ ^]^ Prior research has shown that UL46, a protein encoded by pseudorabies virus (PRV), undergoes crotonylation, yet the functional consequences of this modification remain unclear.^[^ ^51^ ^]^ In this study, we found that MPXV I3 protein undergoes crotonylation. This modification drives I3‐regulated aerobic glycolysis. Interestingly, crotonylation of different proteins plays diverse roles in modulating aerobic glycolysis. For instance, the crotonylation of Aldolase C, which is mediated by glutaryl‐CoA dehydrogenase, inhibits its enzymatic activity, thereby suppressing aerobic glycolysis.^[^ ^52^ ^]^ In contrast, the crotonylation of polypyrimidine tract binding protein 1 promotes aerobic glycolysis by interacting with hnRNPA1/2 and affecting the alternative splicing of PKM.^[^ ^53^ ^]^ Our findings demonstrate that crotonylation of I3 can augment aerobic glycolysis. Thus, our study unveils a novel viral protein target of crotonylation and expands the repertoire of crotonylation‐related factors that participate in aerobic glycolysis.

MYST1, a multifunctional acetyltransferase also known as KAT8 or MOF, possesses an amino‐terminal chromatin domain and a central MYST histone acetyltransferase domain.^[^ ^54^ ^]^ MYST1 has been demonstrated to catalyze crotonylation at various histone sites. Notably, its yeast homolog, ESA1 (essential Sas2‐related acetyltransferase), is responsible for extensive histone crotonylation in yeast,^[^ ^31^ ^]^ indicating that MYST1 functions as an evolutionarily conserved crotonyltransferase. In the current study, we observed an increase in MYST1 expression during MPXV infection and found that MYST1 binds to I3 and mediates its crotonylation. Knockout of MYST1 resulted in the inhibition of aerobic glycolysis and MPXV replication. More significantly, pharmacological inhibition of MYST1 using MC4033 markedly suppressed aerobic glycolysis and viral replication. It is worth noting that 2‐DG treatment further inhibited MPXV replication in MYST1‐deficient cells, implying that beyond MYST1, additional upstream regulatory factors may contribute to the enhanced aerobic glycolysis during MPXV replication. Our findings identify I3 as the first viral protein substrate of MYST1‐catalyzed crotonylation, underscoring its role in aerobic glycolysis and viral replication, and suggesting MYST1 as a potential antiviral target for combating MPXV infection.

Several studies have reported the involvement of MYST1 in viral replication. For instance, MYST1 silencing has been demonstrated to inhibit HBV replication.^[^ ^55^ ^]^ In addition, MYST1 facilitates HIV reactivation by enhancing acetylation of Lys 16 of histone H4.^[^ ^56^ ^]^ Moreover, MYST1 selectively inhibits antiviral immunity by acetylating interferon regulatory factor 3,^[^ ^57^ ^]^ which may facilitate viral replication. In this study, we demonstrate that MYST1 regulates MPXV replication through modulating the crotonylation of viral I3 protein and influencing aerobic glycolysis. These findings expand the known functions and mechanisms of MYST1 in the regulation of viral replication and suggest that its enzymatic activity may play a broader role in viral replication.

WDR26 (WD‐repeat protein 26), a multifunctional cytoplasmic adaptor protein containing WD40 repeats, plays critical roles in diverse cellular processes. These include suppressing MAPK signaling, promoting nuclear condensation during erythropoiesis, decreasing protein stability through CTLH (C‐terminal to LisH) E3 ligase complex interactions, and facilitating mitophagy through control of Parkin translocation.^[^ ^58^, ^59^, ^60^, ^61^ ^]^ Here, we observed increased WDR26 levels in both MPXV‐infected cells and I3‐expressed cells. The binding of crotonylated I3 to WDR26 protected WDR26 from ubiquitination‐dependent degradation. Silencing of WDR26 weakened I3‐induced aerobic glycolysis and markedly impaired MPXV replication. This study represents the first demonstration of WDR26's involvement in aerobic glycolysis and viral replication. However, the underlying molecular mechanisms require further investigation. Specifically, a deeper understanding is needed of the precise mechanisms by which WDR26 modulates aerobic glycolysis and consequently influences viral replication, as well as the identification of the ubiquitination sites on WDR26 and the E3 ubiquitin ligases or deubiquitinating enzymes (DUBs) involved in the crotonylated I3‐mediated suppression of WDR26 ubiquitination and degradation.

The PI3K/AKT pathway is well‐established in its role to induce aerobic glycolysis.^[^ ^62^, ^63^ ^]^ Studies have revealed that WDR26 acts as an activator of the PI3K/AKT pathway,^[^ ^64^ ^]^ suggesting a plausible mechanism through which WDR26‐mediated pathway activation may drive aerobic glycolysis induction. Additionally, as an integral component of the WDR26‐CTLH or Cullin 4B‐DNA damage‐binding protein 1‐WDR26 E3 ubiquitin‐protein ligase complex, WDR26 plays a regulatory role in modulating the ubiquitination and proteasomal degradation of target proteins.^[^ ^65^, ^66^ ^]^ These findings suggest that WDR26 may also influence aerobic glycolysis by regulating the ubiquitination and stability of key negative regulators involved in this metabolic process.

**Figure 6:**
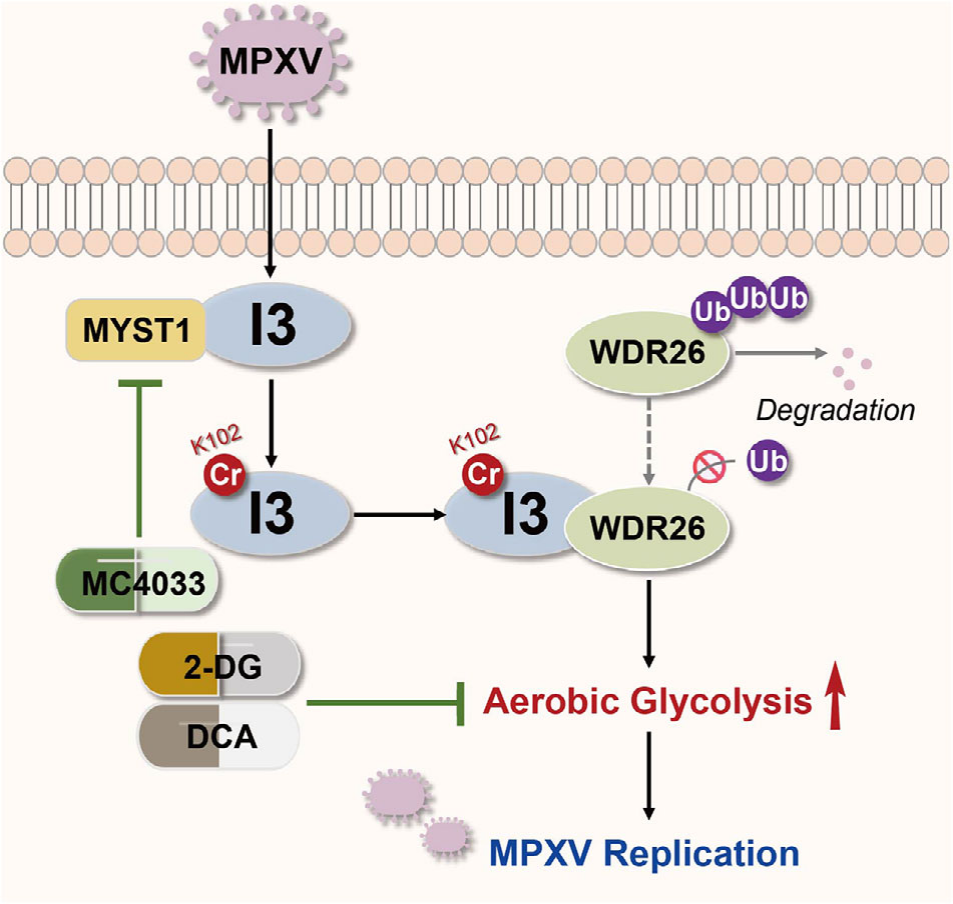
A schematic working model of aerobic glycolysis and viral replication driven by MPXV I3 crotonylation.

In summary, we uncover a novel mechanism by which MPXV exploits aerobic glycolysis to facilitate its replication. Specifically, MPXV infection induces aerobic glycolysis, a process mediated by the viral protein I3 through lysine crotonylation at its K102 residue. The acetyltransferase MYST1 catalyzes the crotonylation of I3 to inhibit the ubiquitin‐mediated degradation of WDR26. Consequently, this process promotes both aerobic glycolysis and viral replication. Targeting either MYST1 or aerobic glycolysis significantly impairs MPXV replication (**Figure** **6**). These findings provide insights into MPXV pathogenesis and identify potential therapeutic targets for combating MPXV infection.

“The MPXV‐encoded I3 protein undergoes MYST1‐mediated crotonylation, which facilitates its interaction with WDR26. Consequently, this interaction reduces WDR26 ubiquitination and degradation, promoting aerobic glycolysis and thereby leading to augmented viral replication. Treatment with MC4033 to target MYST1, with 2‐DG or DCA to inhibit aerobic glycolysis, significantly attenuates MPXV replication. Treatment with MC4033 to target MYST1, with 2‐DG or DCA to inhibit aerobic glycolysis, significantly attenuates MPXV replication.”

## 4 Experimental Section

### Cell Culture

HEK293T (catalog #SCSP‐502), HeLa (catalog #SCSP‐504), and Vero (catalog #SCSP‐520) cells were purchased from Shanghai Cell Bank of the Chinese Academy of Sciences between 2021 and 2023, and were maintained in Dulbecco's Modified Eagle's Medium (DMEM) supplemented with 10% fetal bovine serum (FBS), 100 mg L^−1^ streptomycin, and 100 U mL^−1^ penicillin. The cells were incubated at 37 °C in a 5% CO2 environment. All cell lines were authenticated by short tandem repeat profiling and verified to be mycoplasma‐free using the Mycoplasma Detection Kit from Vazyme Biotech (D103‐01/02, Nanjing, China).

### Reagents and Plasmids

The glycolysis inhibitor 2‐Deoxy‐D‐glucose (2‐DG) (CAS: 154‐17‐6) and MC4033 (CAS: 28532‐21‐0), a selective inhibitor of MYST1, were procured from MedChemexpress (New Jersey, USA). The proteasome inhibitor MG132 (CAS: 1211877‐36‐9), the protein synthesis inhibitor cycloheximide (CHX) (CAS: 66‐81‐9), and the specific inhibitor of PDK, DCA (CAS: 2156‐56‐1) were obtained from Selleck Chemicals (China). The expression plasmids for A9R, A31L, A34L, B6R, B12R, E8L, E12L, H6R, J2R, L3R, OPG030, OPG066, I3, and I3 mutants (K100R/K102R) were constructed using the lentiviral expression vector pCDH‐CMV‐MCS‐EF1‐copGFP (pCDH for short) (Addgene plasmid# 73030, RRID: Addgene_73030) with an HA‐tag (GenScript, Nanjing). Expression plasmids for ESCO1, ESCO2, MYST1, and ATAT1 acyltransferases were created using the pCDH vector with a Myc‐tag, sourced from Sangon Biotech Co., Ltd. (Shanghai).

### Virus Infection

The hMpxV/ChinaGZ8H‐01/2023 strain was maintained at the Changchun Veterinary Research Institute of the Chinese Academy of Agricultural Sciences. All experiments involving MPXV, with a TCID50 of 100, were conducted within the Biosafety Level 3 (BSL‐3) Laboratory of Changchun Veterinary Research Institute of Chinese Agricultural Sciences.

### Lentivirus Packaging and Infection

Lentiviral particles were produced by co‐transfecting HEK293T cells with the packaging plasmid psPAX2 (Addgene plasmid# 12260, RRID: Addgene_12260), the envelope plasmid pMD2.G (Addgene plasmid# 12259, RRID: Addgene_12259), and the lentiviral transfer plasmid pCDH using Lipofectamine 2000. The viral supernatants were collected 48–72 h post‐transfection, filtered through a 0.45 µm membrane, and stored at −80 °C. For infection, lentiviral particles were used in the presence of 10 mg mL^−1^ polybrene to improve infection efficiency.

### Co‐Immunoprecipitation

Cells were washed thrice with ice‐cold PBS and subsequently lysed using IP lysis buffer. After a 10 min incubation on ice, the lysates were gently scraped off, transferred to pre‐chilled microtubes, and incubated for an additional 10 min. Cellular debris was then pelleted by centrifugation at 13 200 rpm for 10 min at 4 °C. Following centrifugation, the supernatant was clarified for verification in the cellular system, and the remaining supernatant was incubated overnight at 4 °C with pre‐cleaned Anti‐tag immunomagnetic beads. ≈20 µL of Anti‐tag immunomagnetic beads were utilized for every 1 × 10^7^ cells. Prior to use, commercial Anti‐tag magnetic beads (Bimake, B26101; B26201; B26301) were washed three times with TBST to remove the preservation solution. The immunoprecipitated complexes were eluted and analyzed by Western blotting or subjected to mass spectrometry (MS). MS analysis was performed by Biotechplc (Beijing, China).

### Western Blotting

The protein levels were assessed by Western blotting as previously described.^[^ ^67^ ^]^ The anti‐GAPDH mouse antibody, and anti‐Tubulin mouse antibody were sourced from Santa Cruz Biotechnology. The anti‐Propionylation rabbit antibody (PTM BIO Cat# PTM‐201, RRID: AB_2904167), anti‐Butyrylation rabbit antibody (PTM BIO Cat# PTM‐301, RRID: AB_2687946), anti‐Crotonylation mouse antibody (PTM BIO Cat# PTM‐502, RRID:AB_2877695), anti‐2‐Hydroxyisobutyrylation mouse antibody (PTM BIO Cat# PTM‐802, RRID:AB_2942100), anti‐β‐Hydroxybutyrylation rabbit antibody (PTM BIO Cat# PTM‐1201, RRID:AB_2927634), anti‐Lactylation rabbit antibody (PTM BIO Cat# PTM‐1401, RRID:AB_2868521), anti‐Malonylation rabbit antibody (PTM BIO Cat# PTM‐901, RRID:AB_2687947), anti‐Glutarylation mouse antibody (PTM BIO Cat# PTM‐1152, RRID:AB_3696874) and anti‐Succinylation mouse antibody (PTM BIO Cat# PTM‐419, RRID:AB_2942099) were obtained from PTM Biolabs (Hangzhou, Zhejiang, China). The Anti‐Flag mouse antibody (MBL International Cat# M185‐3, RRID: AB_10950447), anti‐HA mouse antibody (MBL International Cat# M180‐3, RRID: AB_10951811) and anti‐Myc mouse antibody (MBL International Cat# 562–5, RRID: AB_591116) were purchased from MEDICAL&BIOLOGICAL LABORATORIES CO.LTD. The anti‐MYST1 rabbit antibody (Proteintech Cat# 13842‐1‐AP, RRID:AB_2146894), anti‐YIPF4 rabbit antibody (Proteintech Cat# 15473‐1‐AP, RRID:AB_2217206), and anti‐SERPINB4 rabbit antibody (Proteintech Cat# 11428‐1‐AP, RRID:AB_3085373), anti‐GLUT1 rabbit antibody (Proteintech Cat# 21829‐1‐AP, RRID:AB_10837075), anti‐GLUT1 mouse antibody (Proteintech Cat# 66290‐1‐Ig, RRID: AB_2881673) and anti‐GLUT3 rabbit antibody (Proteintech Cat# 20403‐1‐AP, RRID:AB_10694437) were provided by Proteintech Group, Inc. (Wuhan, China). The anti‐WDR26 rabbit antibody (Huabio Cat# ER1918‐63, RRID: AB_3696873) were provided by HUABIO (Hangzhou, China). Anti‐F3L (Antibody System Cat# PVV14801, RRID: AB_3696877) and anti‐E8L rabbit polyclonal antibody (Antibody System Cat# PVV13201, RRID: AB_3696878) were offered by AntibodySystem (USA). For the generation of I3 polyclonal antibody, the recombinant I3 fragment (amino acids 163–269) was expressed and purified utilizing a prokaryotic expression system. Subsequently, three New Zealand White rabbits were immunized, and the serum was subsequently purified by antigen‐affinity purification.

### RNA Extraction and RT‐qPCR

Total RNA was isolated from cells utilizing TRIzol reagent (Invitrogen, USA) in accordance with the manufacturer's guidelines. Subsequently, reverse transcription was carried out using the HiScript III 1st Strand cDNA Synthesis Kit (Vazyme Biotech Co., Ltd, R312‐01, Nanjing, China). Quantitative real‐time PCR was conducted with ChamQ SYBR qPCR Master Mix (Vazyme Biotech Co., Ltd, Q341‐02, Nanjing, China) on an Applied Biosystems platform (ABI, Foster City, CA, USA). The primer sequences employed are detailed in Table S1 (Supporting Information). GAPDH served as an internal control for normalization purposes.

### Glucose Uptake and Lactate Production Assay

Cells were initially cultured in glucose‐free DMEM for a period of 8 h. Subsequently, the culture medium was replaced, and the cells were incubated with high‐glucose DMEM for an additional 24 h. Following this, the cells were harvested to measure extracellular glucose levels using a Fluorescence‐based Glucose Assay Kit (BioVision, MAK263‐1KT), in accordance with the manufacturer's instructions, as previously described.^[^ ^68^ ^]^ The culture medium was collected to quantify lactate levels using the Lactate Assay Kit (Sangong Biotech Co., Ltd., D799851‐0050, Shanghai) according to the manufacturer's instructions.

### ATP Assessment

ATP levels were measured using the ATP Assay Kit (Beyotime Institute of Biotechnology, S0026, Nantong, China) according to the manufacturer's instructions. Cells were collected, washed, and then ultrasonicated. Post‐centrifugation, the supernatant was collected and assayed for ATP content at an absorbance of 562 nm through Bicinchoninic Acid assay.

### CRISPR‐Cas9 System

The CRISPR‐Cas9 system was utilized to target MYST1, with guide RNAs designed based on a previously published report.^[^ ^69^ ^]^ The MYST1 sgRNA plasmid was generated by inserting the relevant sequences into the LentiCRISPR‐v2 plasmid. Lentiviruses were produced using a packaging system and subsequently used to infect cells. The specific guide RNA sequences employed are provided in Table S2 (Supporting Information).

### Immunofluorescence Assay (IFA)

Cells were seeded overnight on a circular glass coverslip (12 mm diameter) in a 24‐well plate. The cells were then fixed with cold acetone for 15 min, permeabilized with 0.2% Triton X‐100 for 10 min, and blocked with 1% bovine serum albumin for 30 min. Subsequently, they were incubated with the corresponding primary antibody, followed by the corresponding secondary antibody coupled with an Alexa Fluor fluorescent dye. 4′,6‐Diamidino‐2‐phenylindole (DAPI; Beyotime, C1005, China) was then added and incubated for 10 min, and the images were observed using a confocal microscope (Carl Zeiss, Freistaat Thüringen, Germany).

### Plaque Assay

HeLa cells were infected with MPXV for 1 h, after which the culture medium was replaced, and the cells were incubated for another 24 h. Subsequently, the infected cells and supernatants were collected and diluted (10‐fold) in DMEM. Vero cells growing in 12‐well plates were then infected with this diluted viral supernatant. After 2 h of infection, the cells were overlaid with DMEM containing 2% serum and 2% methylcellulose. 72 h post‐infection, the cells were fixed with 4% formaldehyde and stained with 0.2% crystal violet. The number of plaques in each well was counted, and this count was multiplied by the dilution factor to determine the number of plaque‐forming units per unit volume of the original sample.

### Viral Genome Quantification

Total DNA was extracted from the MPXV‐infected cells using the Universal Genomic DNA Kit (CWBIO, CW2298M). Viral genomes were quantified by real‐time PCR using primers specific for the viral F3L gene. The primer sequence for the F3L gene is provided in Table S1 (Supporting Information).

### Nuclear‐Cytoplasmic Separation

Cells were washed twice with prechilled PBS on ice and subsequently lysed in cell lysis buffer (20 mmol L^−1^ Tris‐HCl pH 8.0,0.5%NP40,10 mmol L^−1^ NaCl, 3 mmol L^−1^ MgCl_2_) supplemented with protease inhibitors for 30 min. The lysates were centrifuged at 5000 × g for 5 min at 4 °C to pellet the nuclei, and the supernatant containing cytoplasmic components was collected. The nuclear pellet was washed three times with cell lysis buffer to remove residual cytoplasmic contaminants, followed by lysis in nuclear lysis buffer for 20 min. A brief ultrasonication step was applied to ensure complete nuclear disruption. Nuclear debris was pelleted by centrifugation at 12 500 × g for 10 min at 4 °C, and the supernatant (nuclear extract) was collected. The integrity and purity of the cytoplasmic and nuclear fractions were verified by probing for well‐established marker proteins: GAPDH (cytoplasmic) and LMNB1 (nuclear).

### Statistical Analysis

Data were presented as mean ± SD from at least 3 technical replicates or mean ± SEM from 3 biological replicates as indicated in the figure legends. Two‐sided Student *t*‐test was used for comparisons between two groups. A *p*‐value of 0.05 or less was considered statistically significant. All experiments were repeated at least three times unless otherwise stated.

## Conflict of Interest

The authors declare no conflict of interest.

## Author Contributions

P.W. and Z.Z. contributed equally to this work. P. W., Z. Z., R. X., Q. Y., L. J., F. G., Y. G., T. W. and J. Z. performed the experiments; X. L., Q. Y., C. L. and W. L. conceived the experiments; Q. Y., C. L. and W. L. obtained funding for this work; P. W., Z. Z., R. X., Q. Y., L. J., T. W., C. L., and W. L. conducted formal analysis; P. W., Z. Z., X. L.,Q. Y., C. L., and W. L. performed methodology; P. W. and W. L. wrote the draft of the manuscript; P. W., Q. Y., C. L., and W. L. drew the initial figures and the pattern diagram; Q. Y., C. L., and W. L. reviewed and edited the manuscript.

## Supporting information



Supporting Information

## Data Availability

The data that support the findings of this study are available from the corresponding author upon reasonable request.
