# Supplementary material for: Blocking Lysine Crotonylation and Aerobic Glycolysis as Targeting Strategy Against mpox Virus Replication
Source: Adv Sci (Weinh). 2025 Oct 27;13(4):e09148. doi: 10.1002/advs.202509148 (PMC12822389; doi:10.1002/advs.202509148)
Supplement: Supplementary file 1 — Supporting Information [file ADVS-13-e09148-s001.docx]

Supporting Information

**Blocking lysine crotonylation and aerobic glycolysis as targeting strategy against mpox virus replication**

*Pengjun Wei, ^1 †^ Zongzheng Zhao, ^2 †^ Ruoqi Xu, ^1^ Qin Yan, ^1^ Liangzi Jiang, ^1^ Fuxiao Geng, ^1^ Yang Gu, ^1^ Tianjiao Wang, ^1^ Jing Zhou, ^1^ Xiao Li, ^2^ * Qin Yan, ^1, 3^ * Chun Lu ^1, 3, 4^ * and Wan Li ^1, 3, 4^ **

This file includes:

Figure S1 to S13

Table S1 to Table S2

Supplementary figures


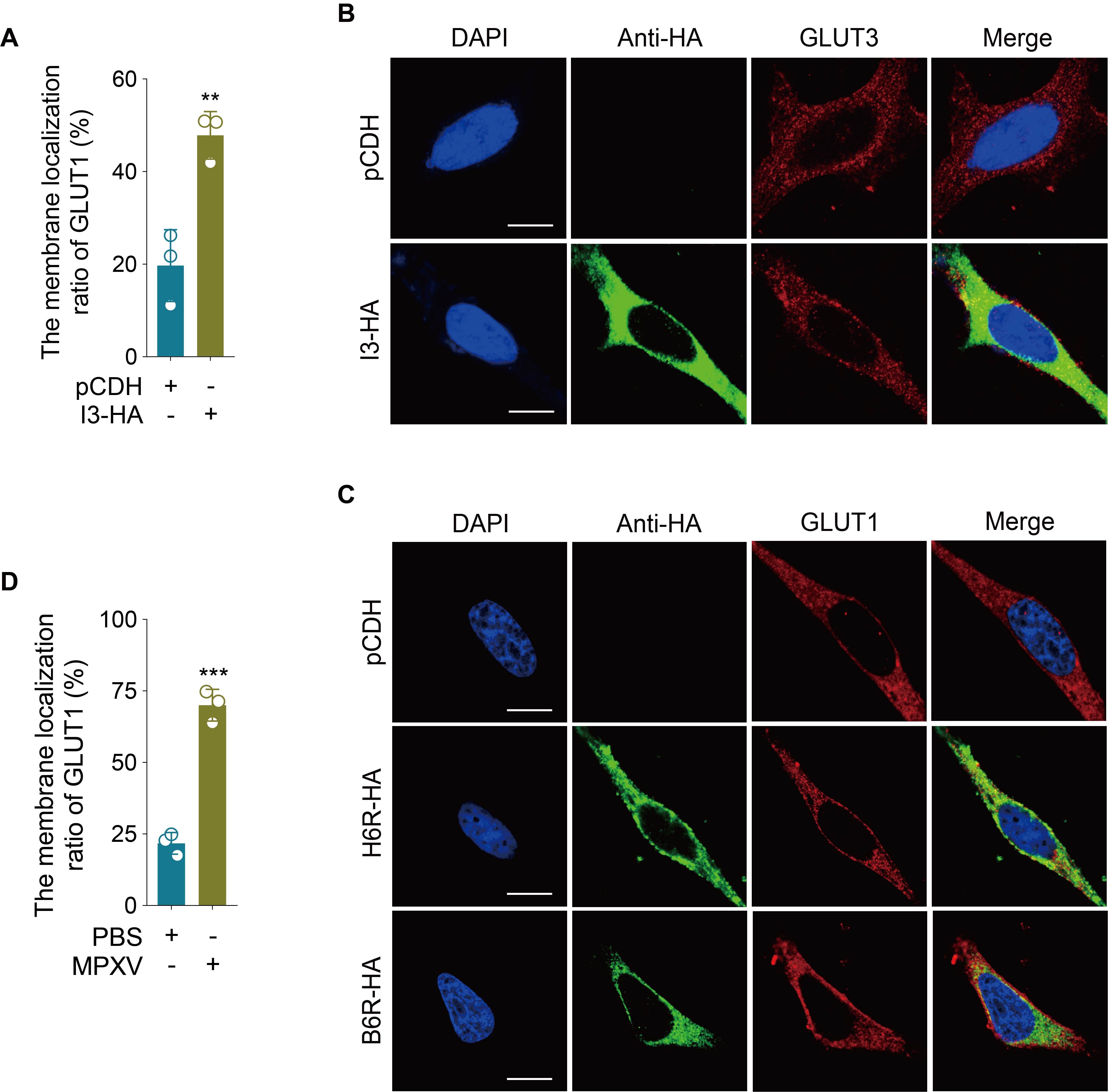


**Figure S1. Detect the intracellular localization of GLUT1 or GLUT3**

**(A)** Results were quantified in (**Figure 1E**) (*n*=3).

**(B)** The localization of GLUT3 in I3-expressing HeLa cells was detected by immunofluorescence staining. The scale bar was 10 μm.

**(C)** The localization of GLUT1 in H6R (**H6R-HA**)- or B6R-expressing (**B6R-HA**) HeLa cells was detected by immunofluorescence staining. The scale bar was 10 μm.

**(D)** Results were quantified in (**Figure 1F**) (*n*=3).

Data are shown as mean ± SD. ** *p* < 0.01, and *** *p* < 0.001, Student's *t*-test.


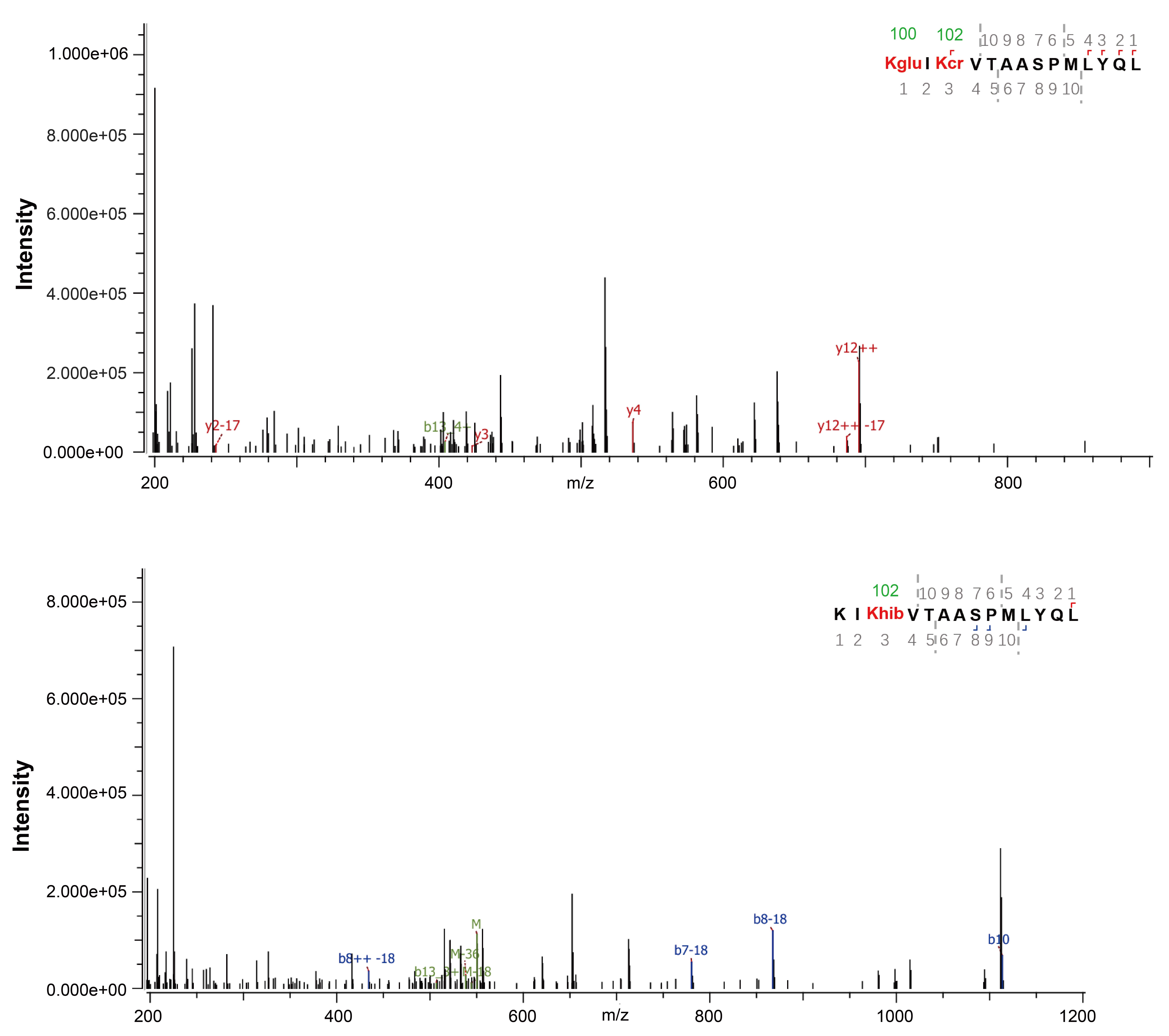


**Figure S2. Mass spectrometry of crotonylation, glutarylation and 2-hydroxybutyrylation sites of I3.**

The crotonylation, glutarylation and 2-hydroxybutyrylation sites of I3 were identified on immunoprecipitated I3-HA using mass spectrometry. Red marks indicate modifications, and green marks indicate sites of modifications.


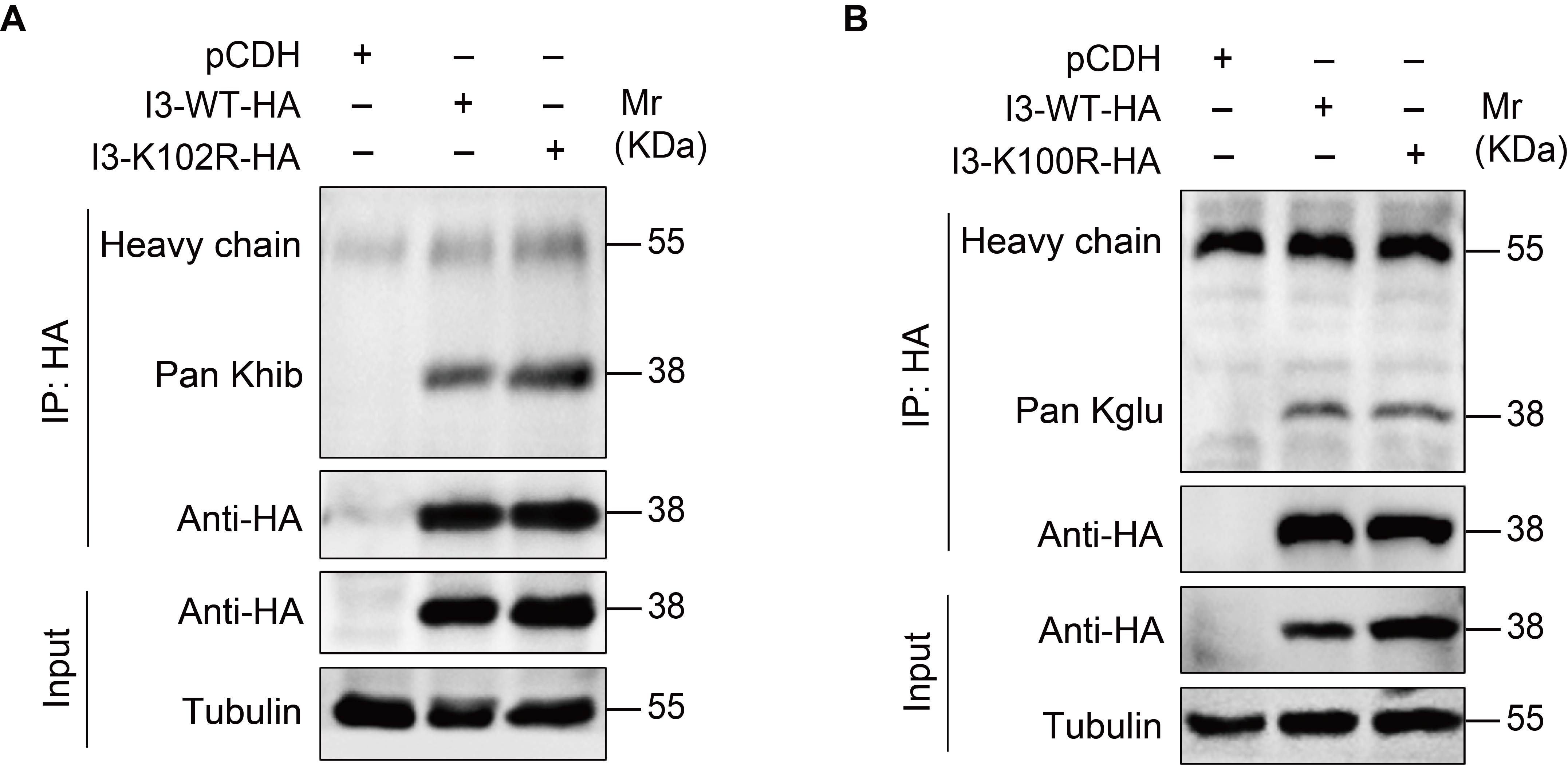


**Figure S3.** **Detecting the levels of glutarylation and 2-hydroxybutyrylation on I3 in cells expressing I3 mutations.**

**(A)** HeLa cells were infected with wild-type I3 (**I3-WT-HA**) or I3 mutant (**I3-K102R-HA**). An IP assay was conducted using an anti-HA antibody to examine the 2-hydroxybutoxylation level of I3.

**(B)** HeLa cells were infected with wild-type I3 (**I3-WT-HA**) or I3 mutant (**I3-K100R-HA**). An IP assay was conducted using an anti-HA antibody to examine the glutarylation level of I3.


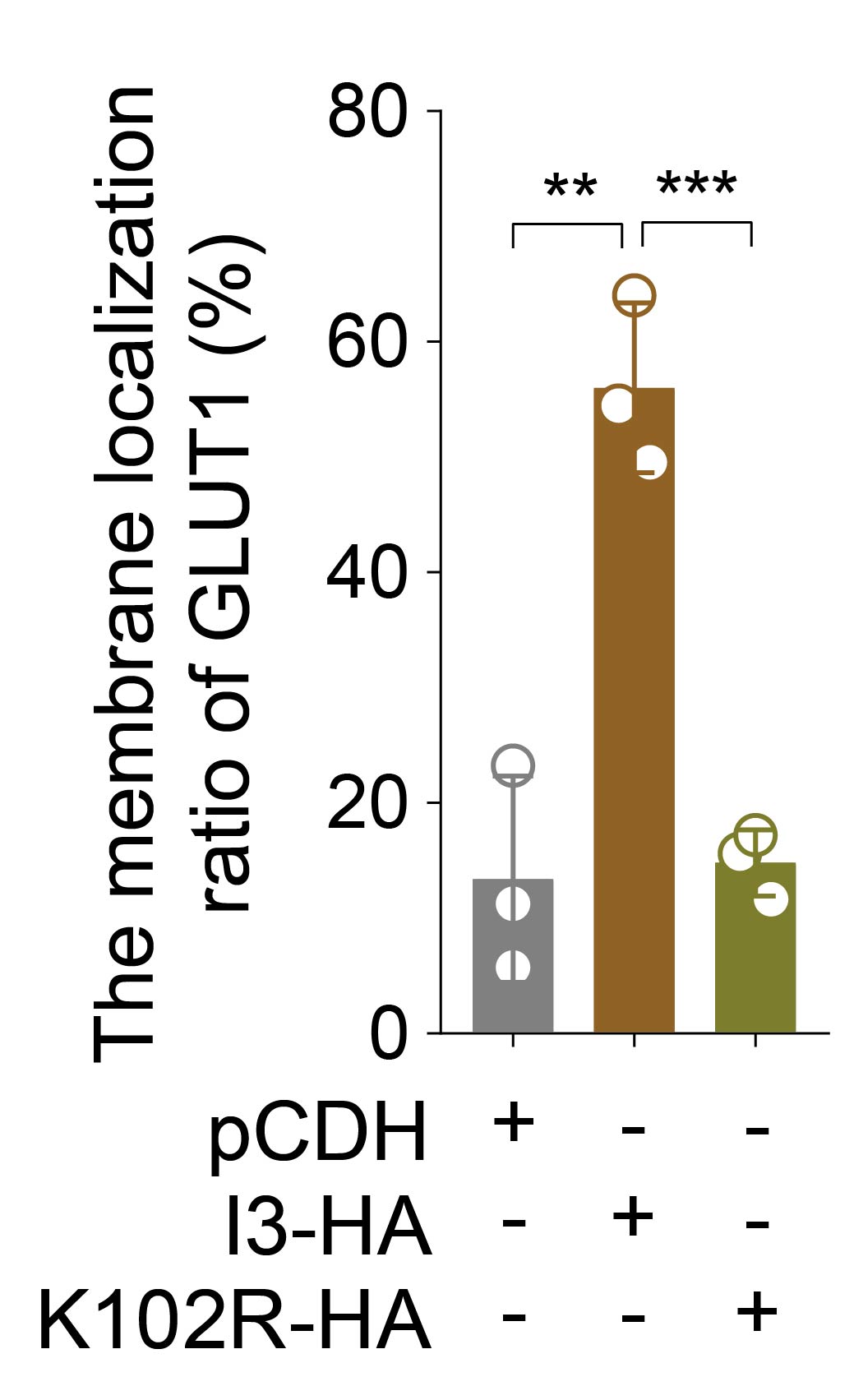


**Figure S4. Crotonylation of I3 at lysine 102 promotes the translocalization of GLUT1.**

Results were quantified in (**Figure 2J**) (*n*=3). Data are shown as mean ± SD. ** *p* < 0.01 and *** *p* < 0.001, Student's *t*-test.


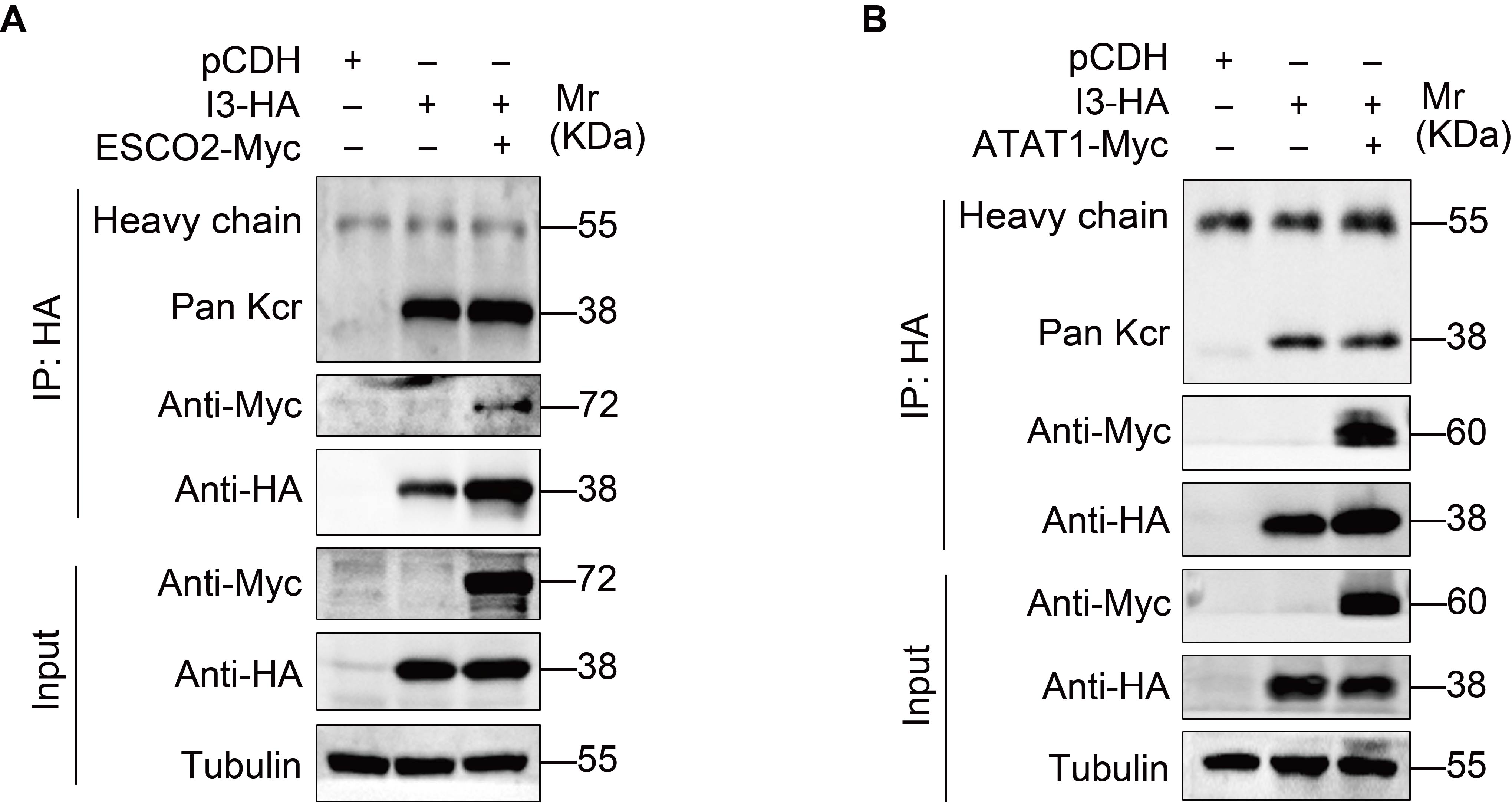


**Figure S5. Effect of ESCO2 and ATAT1 on I3 crotonylation.**

**(A**) HeLa cells transduced with I3 (**I3-HA**) or its control (**pCDH**) were infected with ESCO2 (**ESCO2-Myc**) or its control (**pCDH**). An IP assay was conducted using an anti-HA antibody to examine the crotonylation level of I3.

**(B)** HeLa cells transduced with I3 (**I3-HA**) or its control (**pCDH**) were infected with ATAT1(**ATAT1-Myc**) or its control (**pCDH**). An IP assay was conducted using an anti-HA antibody to examine the crotonylation level of I3.


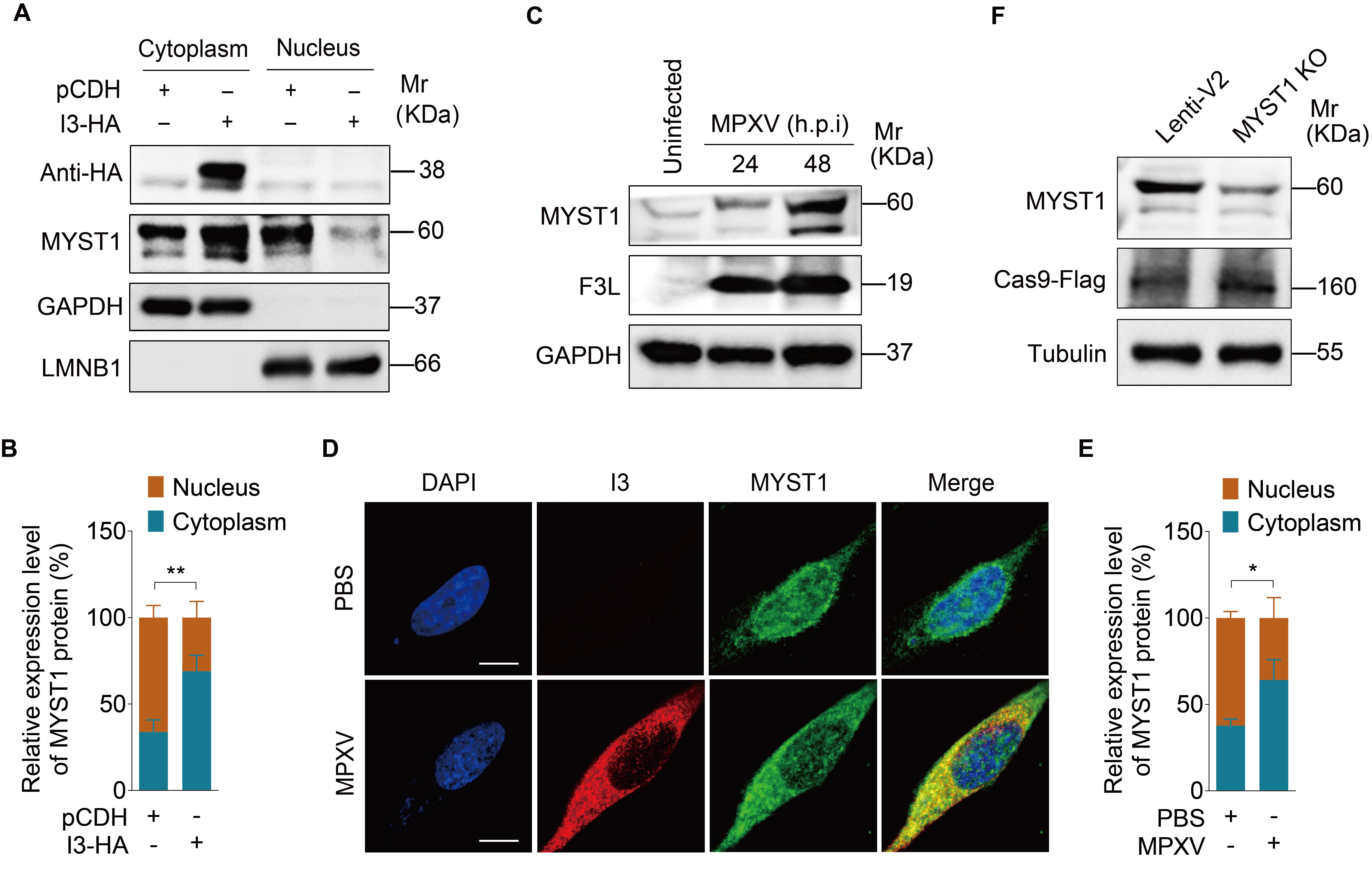


**Figure S6.** **The expression level and the subcellular localization of MYST1 in I3-** **expressing, MPXV-infected and** **MYST1 knockout cells.**

1. Nuclear and cytoplasmic proteins were extracted from HeLa cells expressing I3 (**I3-HA**) or its control (**pCDH**). Western blotting analysis were conducted to examine the distribution of MYST1 in the nucleus or cytoplasm.
2. Results were quantified in (**A**) (*n*=3).

**(C)** HeLa cells infected with MPXV for 24 or 48 h were used to measure the expression level of MYST1.

**(D)** The intracellular localization of I3 and MYST1 in MPXV-infected cells was detected by immunofluorescence staining. The scale bar was 10 μm.

**(E)**  Nuclear and cytoplasmic proteins were extracted from HeLa cells infected by MPXV (**MPXV**) or treated with PBS (**PBS**). Western blotting analysis were performed to measure the distribution of MYST1 in the nucleus or cytoplasm. Results of MYST1 localization were quantified (*n*=3).

**(F)** CRISPR-Cas9 technology was used to construct sgRNA targeting MYST1, and then the lentivirus sgRNA was transfected into HeLa cells. The MYST1-knockout monoclonal cells were obtained after sorting by flow cytometry and screening, and then the level of MYST1 was detected by Western blot analysis.

Data are shown as mean ± SD. * *p* < 0.05 and ** *p* < 0.01, Student's *t*-test.


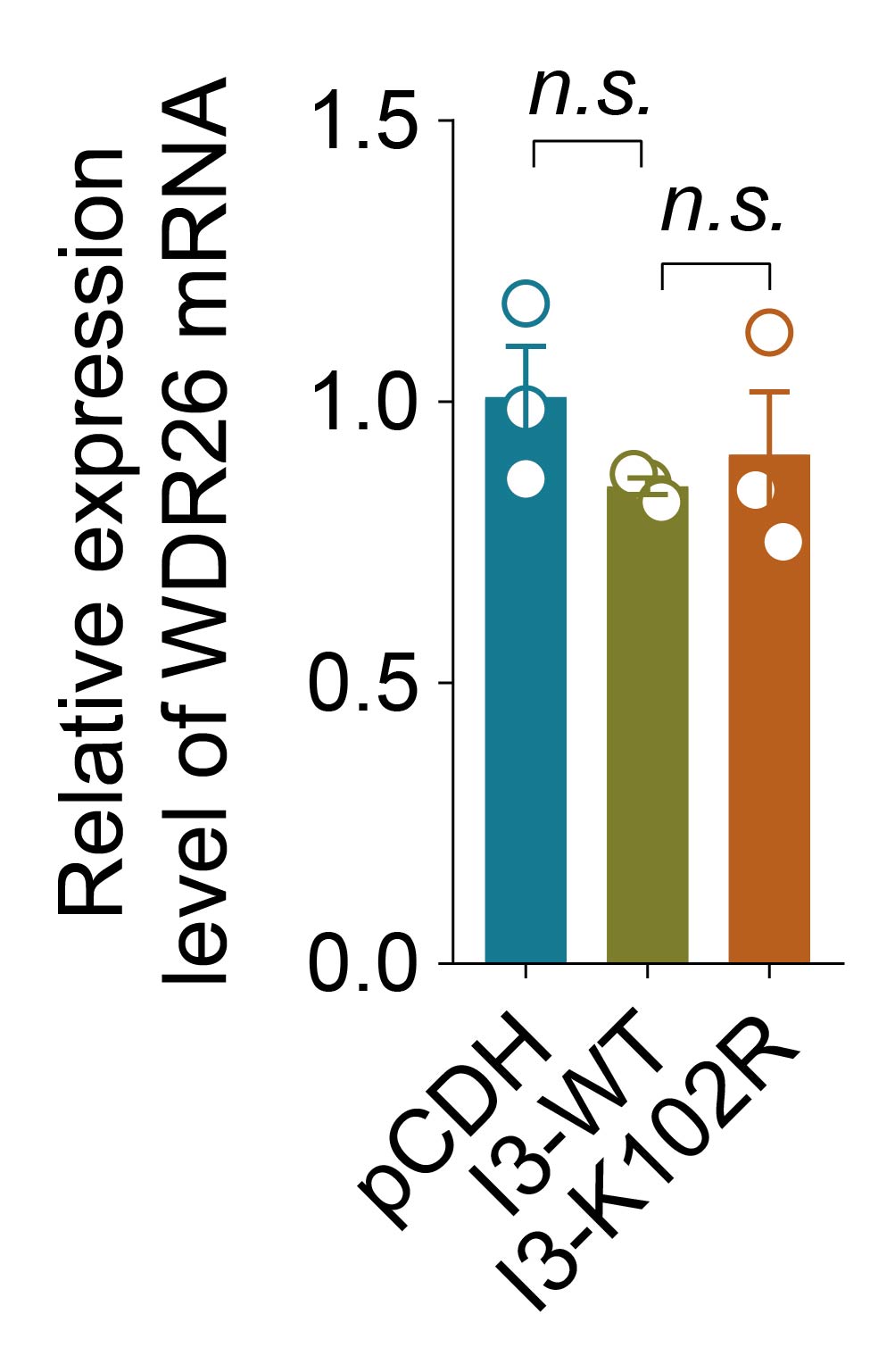


**Figure S7. The expression level of WDR26 mRNA in cells expressing I3 or I3 mutant.**

The mRNA level of WDR26 in HeLa cells transduced with wild-type I3 (**I3-WT-HA**) or I3 mutant (**I3-K102R-HA**) (*n*=3). Data represented the mean ± SEM of three independent experiments. Student's *t*-test. *n.s.*, not significant.


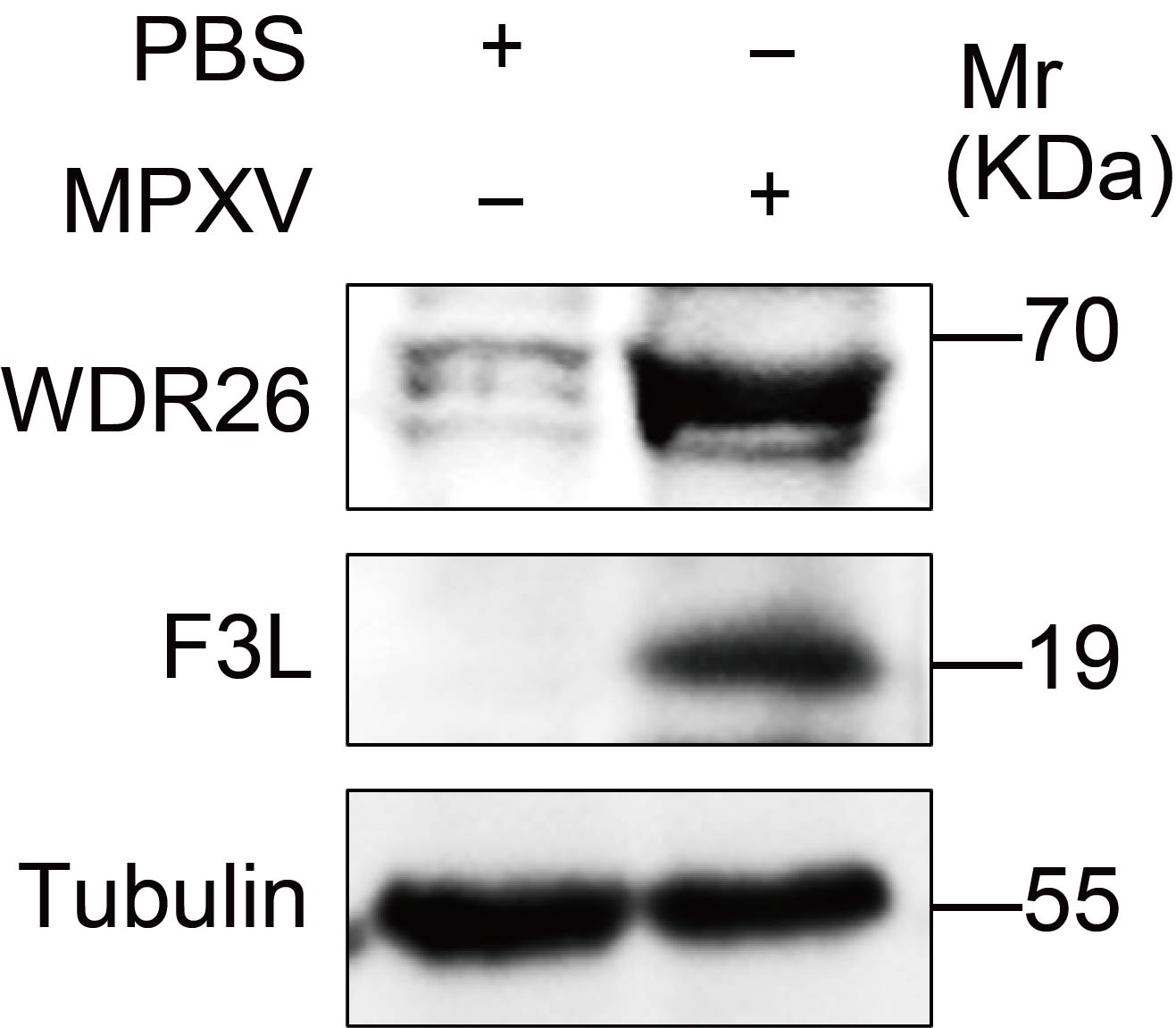


**Figure S8. The expression level of WDR26 in MPXV-infected cells.**

HeLa cells infected with MPXV for 48 h were used to measure the expression level of WDR26.


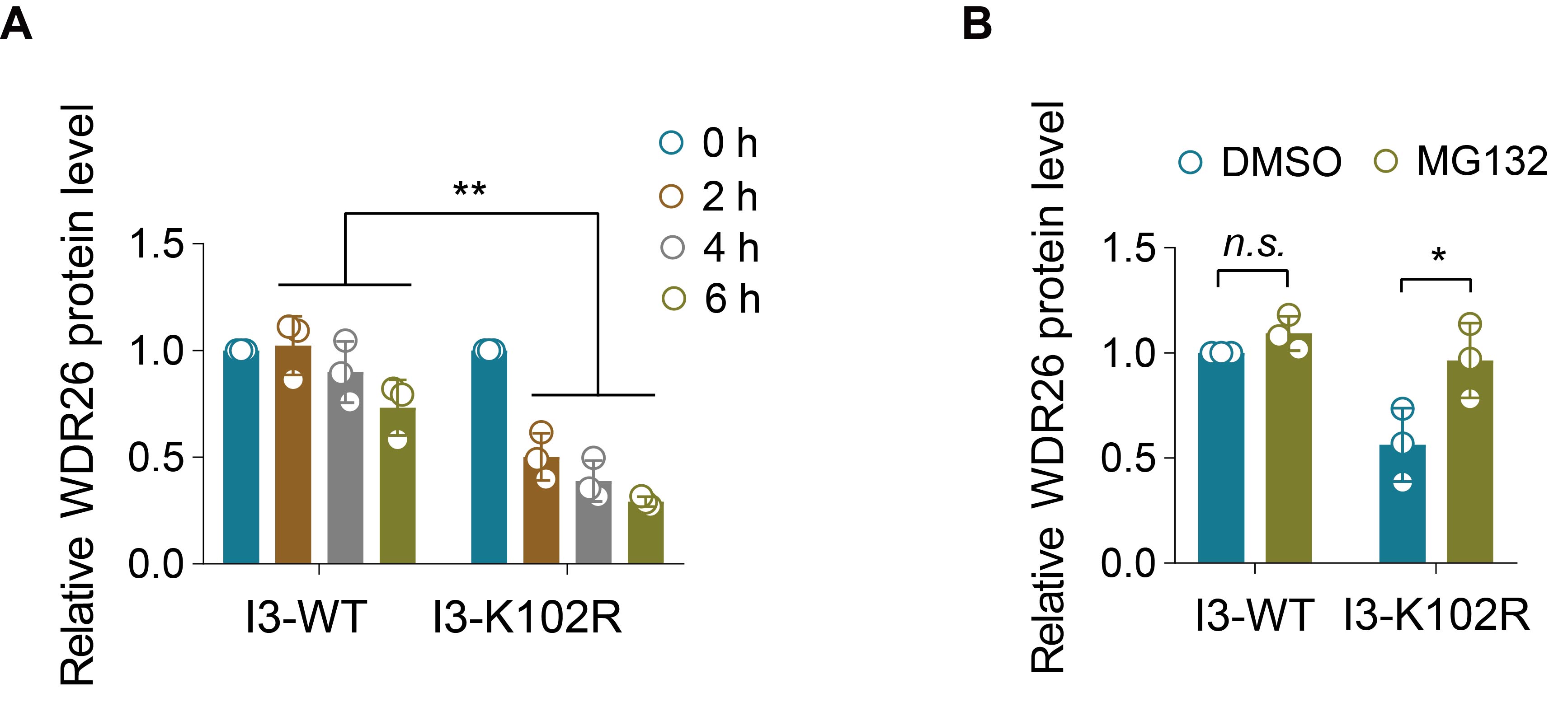


**Figure S9. The quantification of expression level of WDR26.**

**(A)** Results were quantified in (**Figure 4E**) (*n*=3).

**(B)** Results were quantified in (**Figure 4F**) (*n*=3).

Data are shown as mean ± SD. * *p* < 0.05, and ** *p* < 0.01, Student's *t*-test. *n.s.*, not significant.


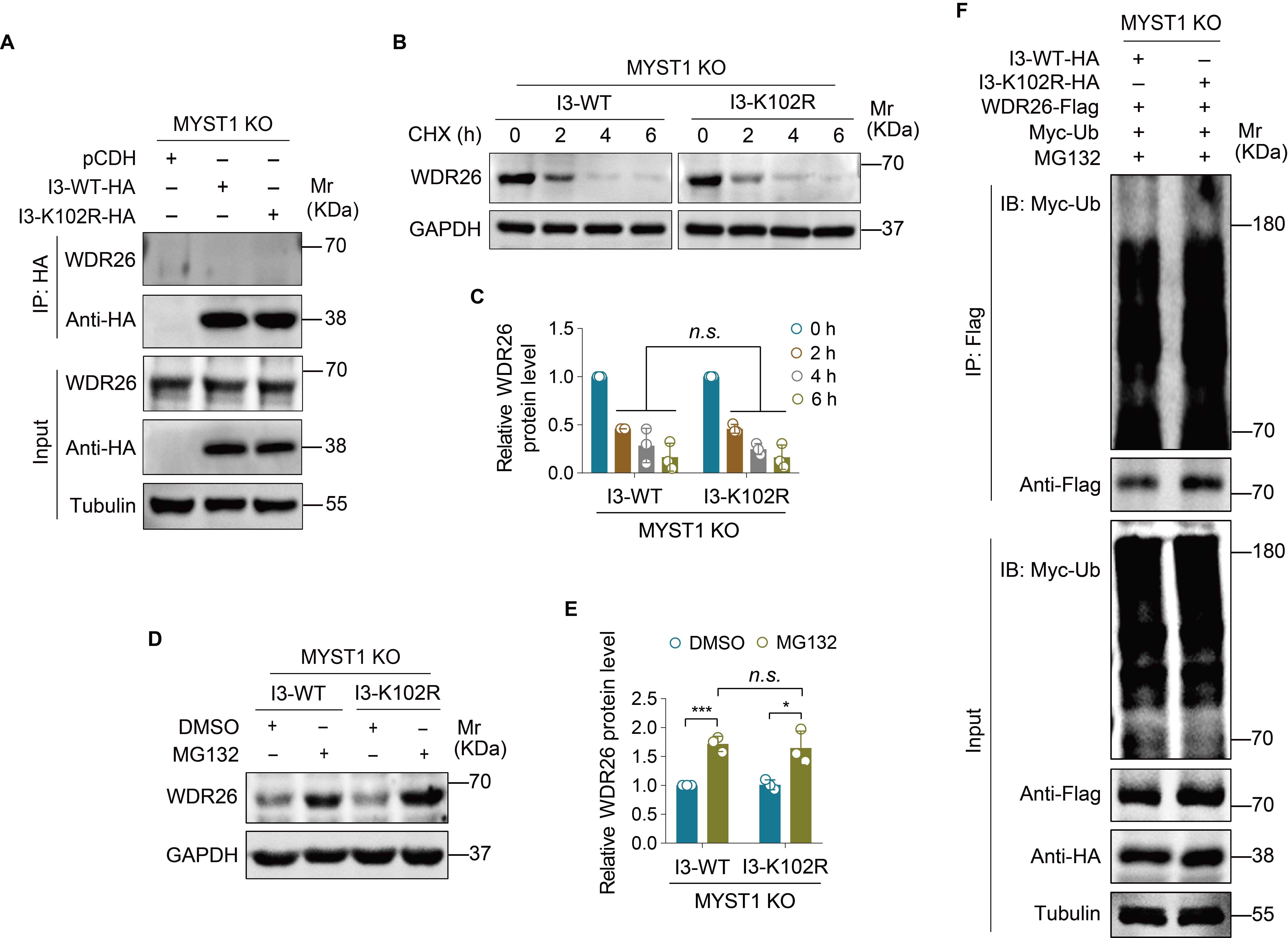


**Figure S10. I3-WT fails to reduce WDR26 ubiquitination and degradation in MYST1** **knockout cells.**

1. MYST1 knockout (**MYST1 KO**) HeLa cells were transduced with wild-type I3 (**I3-WT-HA**) or I3 mutant (**I3-K102R-HA**). An IP assay was performed to assess the interaction between I3-WT or I3-K102R and WDR26.
2. MYST1 knockout (**MYST1 KO**) HeLa cells transduced with wild-type I3 (**I3-WT-HA**) or I3 mutant (**I3-K102R-HA**) were treated with CHX (10 μg/mL) for 0, 2, 4 and 6 h. Western blotting analysis was used to detect the level of WDR26 expression.
3. Results were quantified in (**B**) (*n*=3).
4. MYST1 knockout (**MYST1 KO**) HeLa cells transduced with wild-type I3 (**I3-WT-HA**) or I3 mutant (**I3-K102R-HA**) were treated with MG132 (10 μM) for 2 h. Western blotting analysis was used to detect the level of WDR26 expression.
5. Results were quantified in (**D**) (*n*=3).
6. MYST1 knockout (**MYST1 KO**) HeLa cells transduced with either wild-type I3 (**I3-WT-HA**) or I3 mutant (**I3-K102R-HA**) were subsequently transfected with plasmids encoding Myc-tagged ubiquitin (**Myc-Ub**) and Flag-tagged WDR26 (**WDR26-Flag**). Following transfection, cells were treated with 10 μM MG132 for 2 h. The ubiquitination status of WDR26 was assessed by immunoprecipitation using anti-Flag antibody.

Data are shown as mean ± SD. * *p* < 0.05 and *** *p* < 0.001, Student's *t*-test. *n.s.*, not significant.


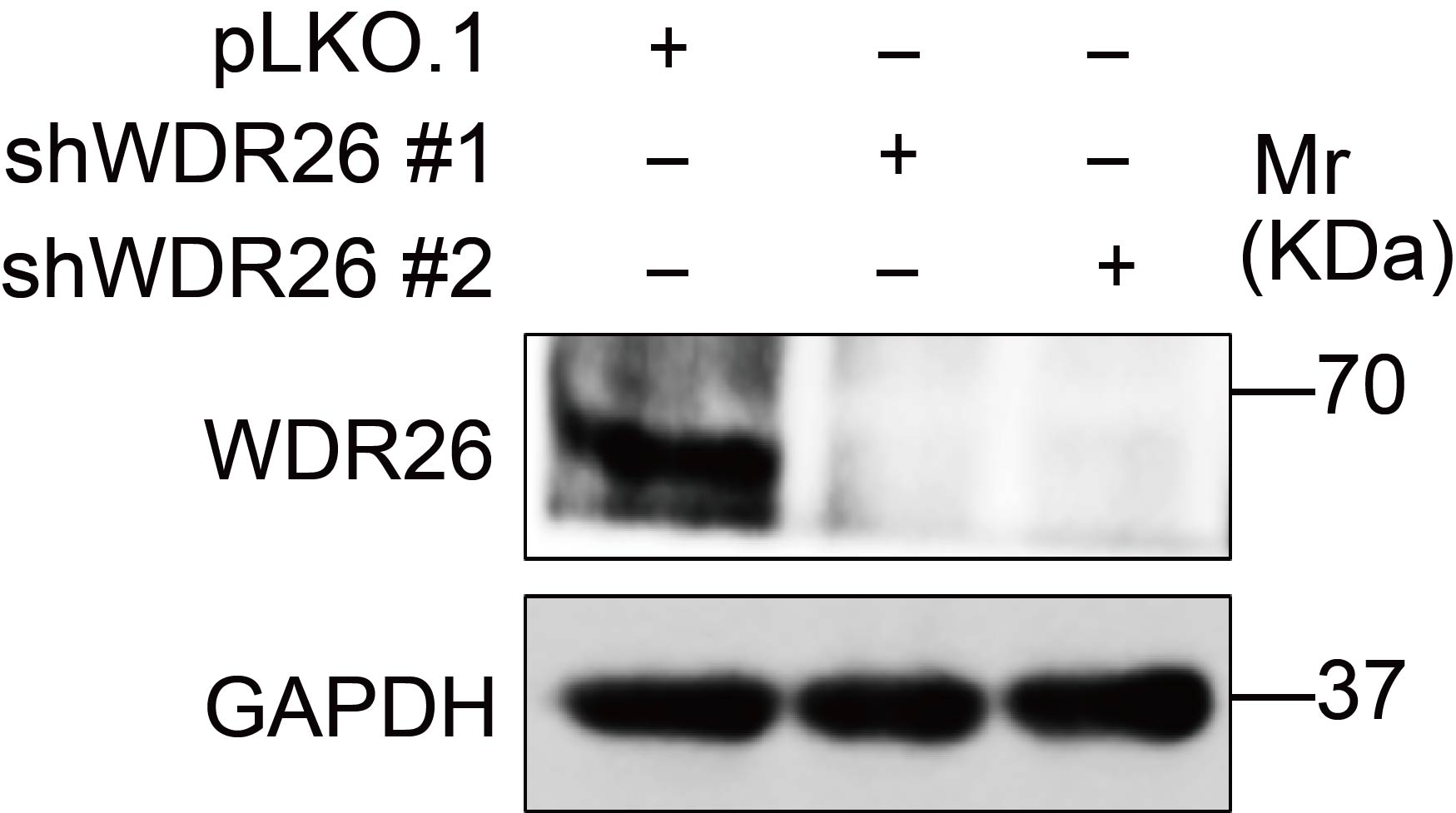


**Figure S11. The expression level of WDR26 in WDR26 knockdown cells.**

HeLa cells transduced with shWDR26 (**shWDR26 #1** and **shWDR26 #2**) or its control (**pLKO.1**), were used for Western blotting analysis to examine the expression level of WDR26.


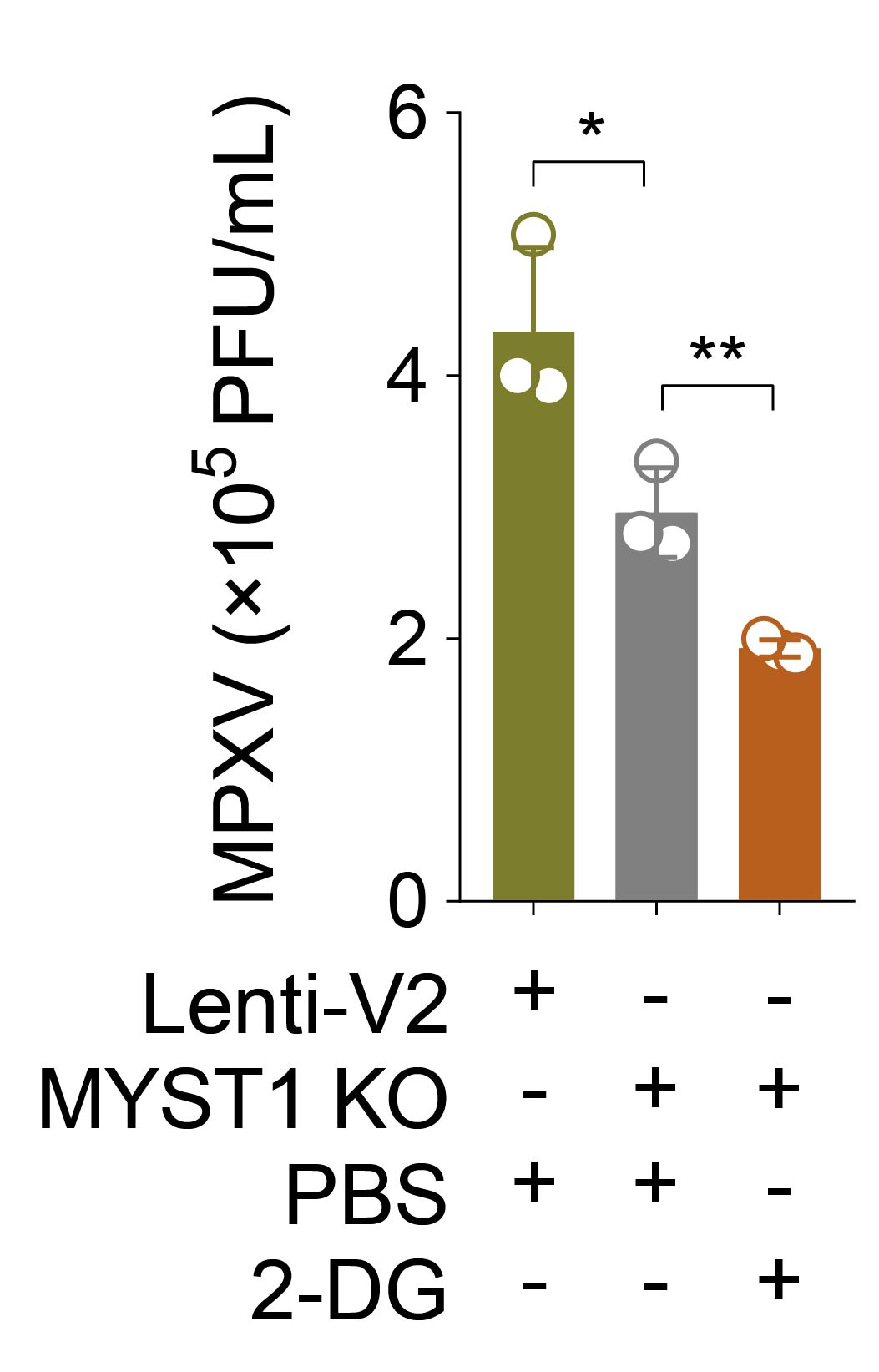


**Figure S12. 2-DG inhibits MPXV replication in MYST1 knockout cells.**

MYST1 knockout (**MTSY1 KO**) HeLa cells were infected with MPXV for 2 h, followed by treating with the glycolysis inhibitor 2-DG (5 mM) for 48 h, and used for conducting a plaque assay (*n*=3). Data are shown as mean ± SD. * *p* < 0.05, and ** *p* < 0.01, Student's *t*-test.


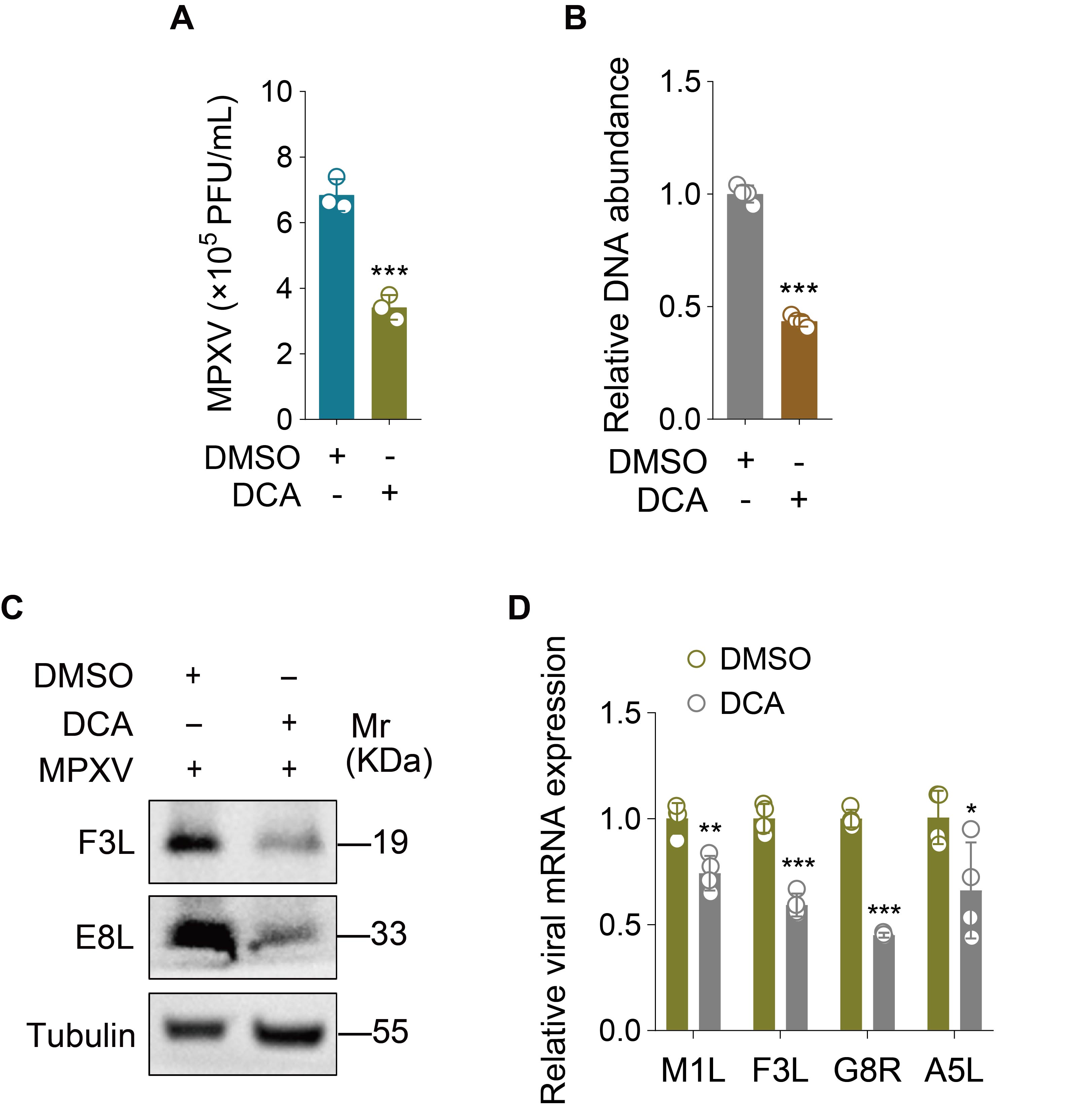


**Figure S13.** **Targeting aerobic glycolysis using DCA** **inhibits MPXV replication.**

1. HeLa cells were infected with MPXV for 2 h, followed by treating with DCA (1 mM) for 48 h, and used for conducting a plaque assay (*n*=3).
2. Cells treated as in (**A**) were used to measure the viral genome copies with viral genome quantification assay (*n*=4).

**(C)** Cells treated as in (**A**) were used to measure the viral protein levels of MPXV (F3L and E8L).

**(D)** Cells treated as in (**A**) were used to examine the mRNA level of MPXV genes (M1L, F3L, G8R and A5L) (*n*=4).

Data are shown as mean ± SD. * *p* < 0.05, ** *p* < 0.01 and *** *p* < 0.001, Student's *t*-test.

**Table S1. The primers for RT-qPCR used in this study**

| **Target** | **Primer** |
| --- | --- |
| F3L | F: 5′- CATCTATTATAGCATCAGCATCAGA -3′ |
|  | R: 5′- GATACTCCTCCTCGTTGGTCTAC -3′ |
| M1L | F: 5′- AACGGACCACATCCTTCTTC -3′ |
|  | R: 5’- ATCCAAACGCGTGTGATAAA-3′ |
| G8R | F: 5′- GCGGATCTGTAAACATTTGG -3′ |
|  | R: 5′- CCTTGGACACTGGAAGGTTAAA -3′ |
| A5L | F: 5′- CTTCCATCCGATTGTTGTGT -3′ |
|  | R: 5′- AGTACACTCCTTCCAGCGTT -3′ |
| GAPDH | F: 5′- GAAGGTGAAGGTCGGAGTC -3′ |
|  | R: 5′- GAAGATGGTGATGGGATTTCC -3′ |
| WDR26 | F: 5′- GAATGGCTTAGGGCTCAACCA -3′ |
|  | R: 5′- TTCTGCCTTATCCCAGTCTCC -3′ |

F: forward; R: reverse

**Table S2. The sequences of the sgRNAs and the shRNAs.**

| **Target** | **Sequence** |
| --- | --- |
| MYST1-sgRNA | AGCCTTGGAGAAGGAGCATGAGG |
| WDR26-shRNA-1 | CCGGCCAGATGACAACTATCTTGTTCTCGAGAACAAGATAGT  TGTCATCTGGTTTTTT |
| WDR26-shRNA -2 | CCGGGCAACAGCCTGAATGTCAATACTCGAGTATTGACATTC  AGGCTGTTGCTTTTTT |
